# Heterologous avian system for quantitative analysis of Syncytin-1 interaction with ASCT2 receptor

**DOI:** 10.1186/s12977-021-00558-0

**Published:** 2021-06-22

**Authors:** Kryštof Štafl, Martin Trávníček, Dana Kučerová, Ľubomíra Pecnová, Veronika Krchlíková, Eliška Gáliková, Volodymyr Stepanets, Jiří Hejnar, Kateřina Trejbalová

**Affiliations:** 1 Institute of Molecular Genetics, Czech Academy of Sciences, Vídeňská 1083, 14220 Prague 4, Czech Republic; 2 Faculty of Science, Charles University, Albertov 6, 12800 Prague 2, Czech Republic

**Keywords:** Retroviral receptor, Envelope glycoprotein, Envelope-receptor interaction, ASCT2 (SLC1A5), Syncytin-1, Cell–cell fusion, NanoLuc luciferase

## Abstract

**Background:**

Human Syncytin-1 is a placentally-expressed cell surface glycoprotein of retroviral origin. After interaction with ASCT2, its cellular receptor, Syncytin-1 triggers cell–cell fusion and formation of a multinuclear syncytiotrophoblast layer of the placenta. The ASCT2 receptor is a multi-spanning membrane protein containing a protruding extracellular part called region C, which has been suggested to be a retrovirus docking site. Precise identification of the interaction site between ASCT2 and Syncytin-1 is challenging due to the complex structure of ASCT2 protein and the background of endogenous *ASCT2* gene in the mammalian genome. Chicken cells lack the endogenous background and, therefore, can be used to set up a system with surrogate expression of the ASCT2 receptor.

**Results:**

We have established a retroviral heterologous chicken system for rapid and reliable assessment of ectopic human ASCT2 protein expression. Our dual-fluorescence system proved successful for large-scale screening of mutant ASCT2 proteins. Using this system, we demonstrated that progressive deletion of region C substantially decreased the amount of ASCT2 protein. In addition, we implemented quantitative assays to determine the interaction of ASCT2 with Syncytin-1 at multiple levels, which included binding of the soluble form of Syncytin-1 to ASCT2 on the cell surface and a luciferase-based assay to evaluate cell–cell fusions that were triggered by Syncytin-1. Finally, we restored the envelope function of Syncytin-1 in a replication-competent retrovirus and assessed the infection of chicken cells expressing human ASCT2 by chimeric Syncytin-1-enveloped virus. The results of the quantitative assays showed that deletion of the protruding region C did not abolish the interaction of ASCT2 with Syncytin-1.

**Conclusions:**

We present here a heterologous chicken system for effective assessment of the expression of transmembrane ASCT2 protein and its interaction with Syncytin-1. The system profits from the absence of endogenous ASCT2 background and implements the quantitative assays to determine the ASCT2-Syncytin-1 interaction at several levels. Using this system, we demonstrated that the protruding region C was essential for ASCT2 protein expression, but surprisingly, not for the interaction with Syncytin-1 glycoprotein.

**Graphical abstract:**

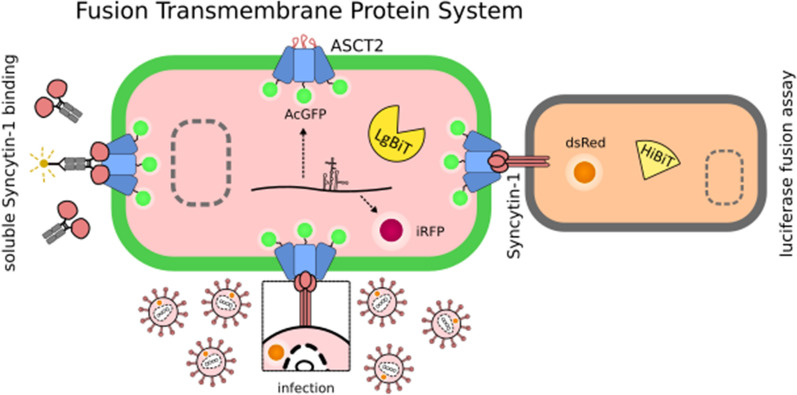

**Supplementary Information:**

The online version contains supplementary material available at 10.1186/s12977-021-00558-0.

## Background

Syncytin-1 was identified as an essential gene implicated in human placenta morphogenesis and function [1, 2]. Specifically, it triggers the cell–cell fusion of cytotrophoblast and formation of multinucleated syncytiotrophoblast. The intrinsic fusogenic function of Syncytin-1 relates to its viral origin. The human *syncytin*-1 gene represents the retroviral *envelope* (*env*) of endogenous ERVW-1 provirus that had been exapted for placentation. To prevent undesirable formation of multinucleated syncytia in non-placental tissues, *syncytin*-1 expression is restricted to the placenta by several mechanisms which include epigenetic modifications of the 5' LTR regulatory region of ERVW-1, availability of specific transcription factors, and effective splicing of env mRNA that occurs exclusively in trophoblast and, aberrantly, in germ cell tumours [3–9]. Once synthesised, the Syncytin-1 protein undergoes post-translational modifications, homo-trimer assembly, and cleavage of its surface (SU) and transmembrane (TM) subunits by cellular furin protease. Finally, Syncytin-1 is transported to the plasma membrane to exert its fusogenic function [10]. Through the receptor-binding domain (RBD), the SU subunit of Syncytin-1 is responsible for binding to the specific cellular receptor [11]. This interaction leads to Syncytin-1 conformational rearrangements that drive the membrane fusion process and multinucleated syncytium formation.

**Fig. 1:**
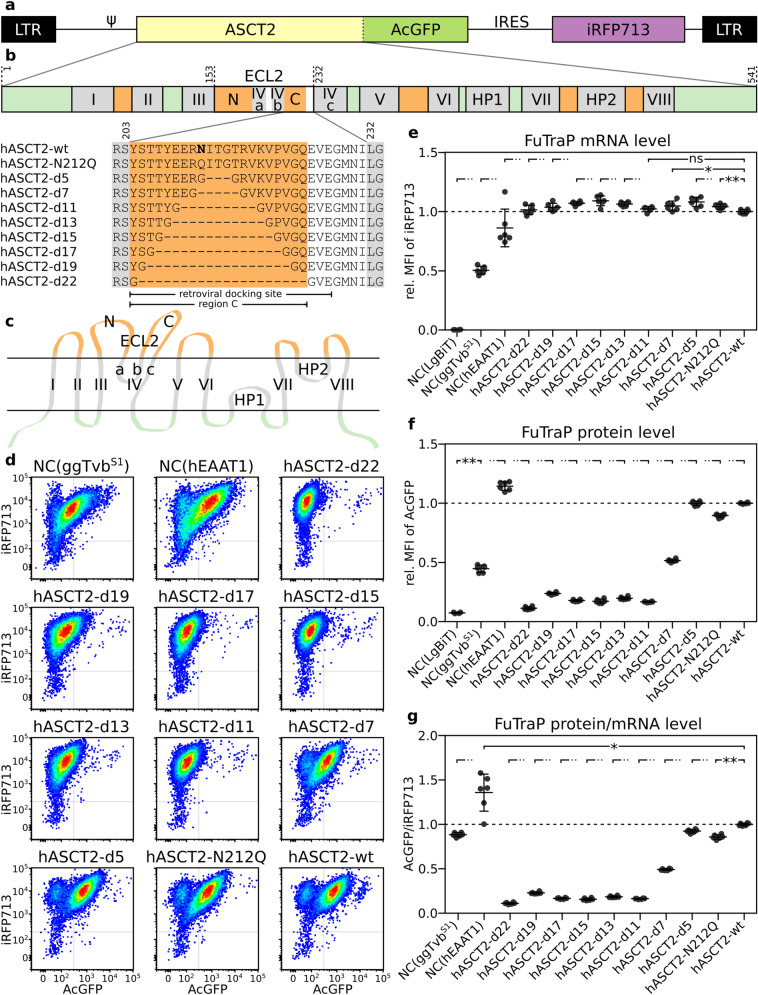
pFuTraP vectors and their expression. **a** Scheme of the pFuTraP-hASCT2-wt vector. The vector was constructed using MAV LTRs, RCASBP encapsidation signal (Ψ), human ASCT2 fused with AcGFP, IRES, and iRFP713 fluorescent marker. **b** Scheme of the ASCT2 structure and alignment of mutants. Depicted are eight transmembrane helices and two helical hairpins (displayed in grey, numbered I-VIII and HP1-2, respectively), which divide the protein into intracellular (light green) and extracellular parts (orange). The longest extracellular loop (ECL2) is interrupted by short membrane regions (IVa and IVb) to the N-terminal (region N) and C-terminal (region C) parts. Region C is stabilised by two antiparallel beta-sheets and was proposed as a retroviral docking site. The amino acid sequences of ASCT2 mutants with deglycosylated and shortened region C used in this study are shown. The numbering corresponds to the protein sequence of wild-type ASCT2, the glycosylated asparagine N212 is shown in bold. **c** Scheme of the membrane topology of ASCT2 (modified from [26]). **d** iRFP713 and AcGFP expression from pFuTraP vectors. DF-1/LgBiT cells with ectopic expression of pFuTraP vectors were characterised by flow cytometry. The analysis was repeated three times in biological duplicates and representative dot plots of 3 × 10^4^ cells are presented. The X-axis depicts the AcGFP fluorescence, Y-axis depicts the iRFP713 fluorescence. **e** iRFP713 fluorescence reflects the level of RNA produced from pFuTraP vectors. The MFI of iRFP713 of individual variants relative to the FuTraP-hASCT2-wt is plotted. **f** AcGFP fluorescence reflects the level of FuTraP protein produced from pFuTraP vectors. The MFI of AcGFP of individual variants relative to the FuTraP-hASCT2-wt is plotted. **g** Protein production from pFuTraP vectors. For each sample, the relative MFI of AcGFP (protein level) was normalised to the relative MFI of iRFP713 (mRNA level). NC, negative control (ggTvb^S1^ or hEAAT1). Data is shown as means ± standard errors, *p < 0.05, **p < 0.01, Mann–Whitney two-tailed test

Two sodium-dependent neutral amino acid transporters, ASCT2 (SLC1A5) and, alternatively, ASCT1 (SLC1A4), were identified as Syncytin-1 cellular receptors [1, 12]. Both proteins are widely expressed, including human placenta. Besides its role in trophoblasts fusion, ASCT2 as a cellular glutamine transporter was found to be overexpressed in various human tumours and is related to poor prognosis [13–15]. The ASCT2 gene is present in most vertebrates, but importantly, it is missing in the chicken genome [16]. ASCT2 serves as receptor for the entire RD114-and-D-type-retrovirus (RDR) interference group, which is comprised of distinct retroviruses isolated from different mammalian and avian hosts [17–20]. Furthermore, the RDR-related envs exploiting ASCT2 as a specific receptor were identified in the genomes of several mammalian endogenous retroviruses [21–23]. ASCT2 is organised as a homo-trimeric multi-membrane spanning protein with each monomer consisting of five extracellular loops (ECLs). ECL2 of ASCT2 folds into two extracellular parts that are separated by a short in-membrane region (Fig. 1c). The C-terminal part of ECL2, designated as region C, has been suggested to be critical for the interaction with Syncytin-1 [24]. Cryo-electron microscopy of ASCT2 showed that region C protrudes from the cell surface into the extracellular space, and hence has been proposed as a retrovirus docking site [25, 26]. Furthermore, N-glycosylation at two sites within ECL2 has been shown to change the receptor affinity to envelope glycoproteins as well as receptor transport to the plasma membrane [12, 24, 27]. Nevertheless, despite intensive study, the specific ASCT2 amino acid residues involved in the interaction with Syncytin-1 have not been identified.

Our understanding of the Syncytin-1-receptor interaction is complicated by the availability of two alternative receptors (ASCT1 and ASCT2) in mammalian cells and difficulties with manipulating integral transmembrane proteins. To define the molecular determinants of Syncytin-1 and its cellular receptor interaction, advanced systems that precisely monitor the receptor are highly required.

In this study, we present such a methodological approach focused on ASCT2 as a proof of concept. We developed a novel dual-fluorescence system, FuTraP (Fusion Transmembrane Protein), for ectopic expression of human ASCT2 in chicken cells. To determine the ASCT2-Syncytin-1 interaction, we implemented several quantitative assays that allowed us to evaluate the binding of the soluble form of Syncytin-1 to ASCT2 and assess cell infection with a replication-competent reporter retrovirus with the Syncytin-1 envelope. Additionally, we developed a new quantitative assay of cell–cell fusion based on a complementation of two-component luciferase. We have demonstrated that our chicken heterologous FuTraP system represents an efficient tool to study the interaction of Syncytin-1 and ASCT2 receptor.

## Results

### Avian retroviral vector pFuTraP expressing ASCT2

For ectopic expression of wild-type and mutated ASCT2, we chose to use the chicken cell system, which provided several advantages. i. The absence of the ASCT2 gene in the chicken genome [16] eliminates any background of endogenous ASCT2 gene product and its interference with the ectopically expressed protein. ii. Efficient ectopic expression of ASCT2 can be driven by a versatile avian replication-defective retroviral vector, which stably integrates in the chicken cell genome. iii. The interaction of Syncytin-1 with ASCT2 can be simulated by entry of a chimeric avian leukosis-based virus (ALV) carrying the Syncytin-1 envelope. iv. ASCT2-expressing and Syncytin-1-enveloped retroviral vectors can be easily produced in previously prepared chicken packaging cells and in the DF-1 chicken cell line, respectively.

We constructed a dual-fluorescence ASCT2 expression vector, named here as pFuTraP-hASCT2-wt, that contains the wild-type human ASCT2 fused to the Green Fluorescent Protein from *Aequorea coerulescens* (AcGFP), followed by an IRES-iRFP713 cassette (Fig. 1a, b). For ASCT2 display, we employed DF-1 cells with stable expression of one fragment of split luciferase reporter, the DF-1/LgBiT cells. We transduced chicken DF-1/LgBiT cells with VSV-G-pseudotyped viral particles carrying the FuTraP-hASCT2-wt genome and obtained cells expressing two proteins of the FuTraP system, specifically, the ASCT2-AcGFP fusion protein and the iRFP713 protein. ASCT2-AcGFP permits exact quantification and direct localisation of the ASCT2 protein. iRFP713 translation occurs from the same mRNA as the ASCT2-AcGFP fusion protein but is initiated at the Internal Ribosome Entry Site (IRES) sequence. The fluorescence intensity of iRFP713 thus reflects the mRNA level of the FuTraP transcript and allows us to enrich the successfully modified cells regardless of the ASCT2 expression levels. After the transduction of DF-1/LgBiT cells with the FuTraP-hASCT-wt, we sorted the cell population with efficient expression of the iRFP713 fluorescent protein (see the bottom-right hASCT2-wt dot-plot in Fig. 1d).

Next, to demonstrate the comparative capacity of the pFuTraP-hASCT2 system, we explored the role of region C that has been suggested as a retroviral docking site. We designed pFuTraP-hASCT2-d5, -d7, -d11, -d13, -d15, -d17, -d19, and -d22 mutants containing progressively extended deletions of five to 22 amino acids from the ASCT2 receptor region C (Fig. 1b). The largest deletion removed the entire region C. We also constructed a pFuTraP-hASCT2-N212Q mutant with abolished glycosylation within region C (Fig. 1b).

The coding sequence of human EAAT1 (hEAAT1, also SLC1A3), glutamate and aspartate multi-spanning membrane transporter that is structurally similar to ASCT2 [28], but was not reported to interact with Syncytin-1, was fused with AcGFP, cloned into the pFuTraP vector and used as a negative control (NC). Further, the chicken Tvb^S1^ molecule (ggTvb^S1^), which confers sensitivity to ALV subgroups B, D, and E [29] and contains a single membrane-spanning domain, was fused with AcGFP and inserted into the pFuTraP vector as another non-specific negative control.

Similarly to the wild-type FuTraP-hASCT2-wt, we transduced DF-1/LgBiT cells with the individual FuTraP-hASCT2 mutants and negative controls and sorted the iRFP713-positive cells (Fig. 1d). The mean fluorescence intensity (MFI) of iRFP713-sorted cells was normalised to that of FuTraP-hASCT2-wt and plotted as the relative FuTraP mRNA level. One of the negative controls, FuTraP-ggTvb^S1^, displayed lower relative MFI of iRFP713 (Fig. 1d, e), probably reflecting its cytopathic effect [30]. Importantly, the relative mRNA levels of all FuTraP-hASCT2 variants ranged between 1.09 and 1.00, emphasizing a comparable efficiency of our transduction-based approach (Fig. 1e).

### The ASCT2 protein level affected by deletion of region C

The fluorescence intensity of AcGFP, which was C-terminally fused to the wild-type or mutant receptor, monitored expression of the ASCT2 protein. Since it was difficult to separate the AcGFP-positive and AcGFP-negative populations in some of the mutants (Fig. 1d), we evaluated the MFI of AcGFP for the entire cellular population (Fig. 1f). The MFI of AcGFP was normalised to that of FuTraP-hASCT2-wt and plotted as the relative FuTraP protein level. The MFI of non-fluorescent DF-1/LgBiT cells served as the negative control. The AcGFP fluorescence of all FuTraP-hASCT2 mutants was significantly higher than that of the DF-1/LgBiT negative control (Fig. 1f), including the FuTraP-hASCT2-d22 mutant missing the entire region C. However, we observed that the increasing extent of region C deletion led to a decline in MFI of AcGFP, which indicates a lower level of mutated hASCT2 proteins.

To assess the effect of region C on the protein level of the ASCT2, we calculated the ratio of AcGFP intensity that reflected the level of the protein (Fig. 1f) to the iRFP713 intensity that reflected the mRNA level (Fig. 1e). For example, the normalised protein level of the FuTraP-hASCT2-N212Q glycosylation mutant reached 86% of the wild-type receptor, indicating a mild positive effect of glycosylation on the protein translation and/or stability of ASCT2 (Fig. 1g). Further, we observed that progressive deletion of region C led to a substantial decline of the normalised ASCT2 protein amount when compared to the wild-type ASCT2 (Fig. 1g). The decreased protein level after deletion of region C was verified by Western blotting using an anti-ASCT2 antibody (Additional file 1: Fig. S1a, b). Western blotting thus validated the specificity of our dual fluorescence-based quantitative approach. Our results revealed that region C was essential for the protein expression/stability of ASCT2.

### ASCT2 cell surface localisation and receptor function

**Fig. 2:**
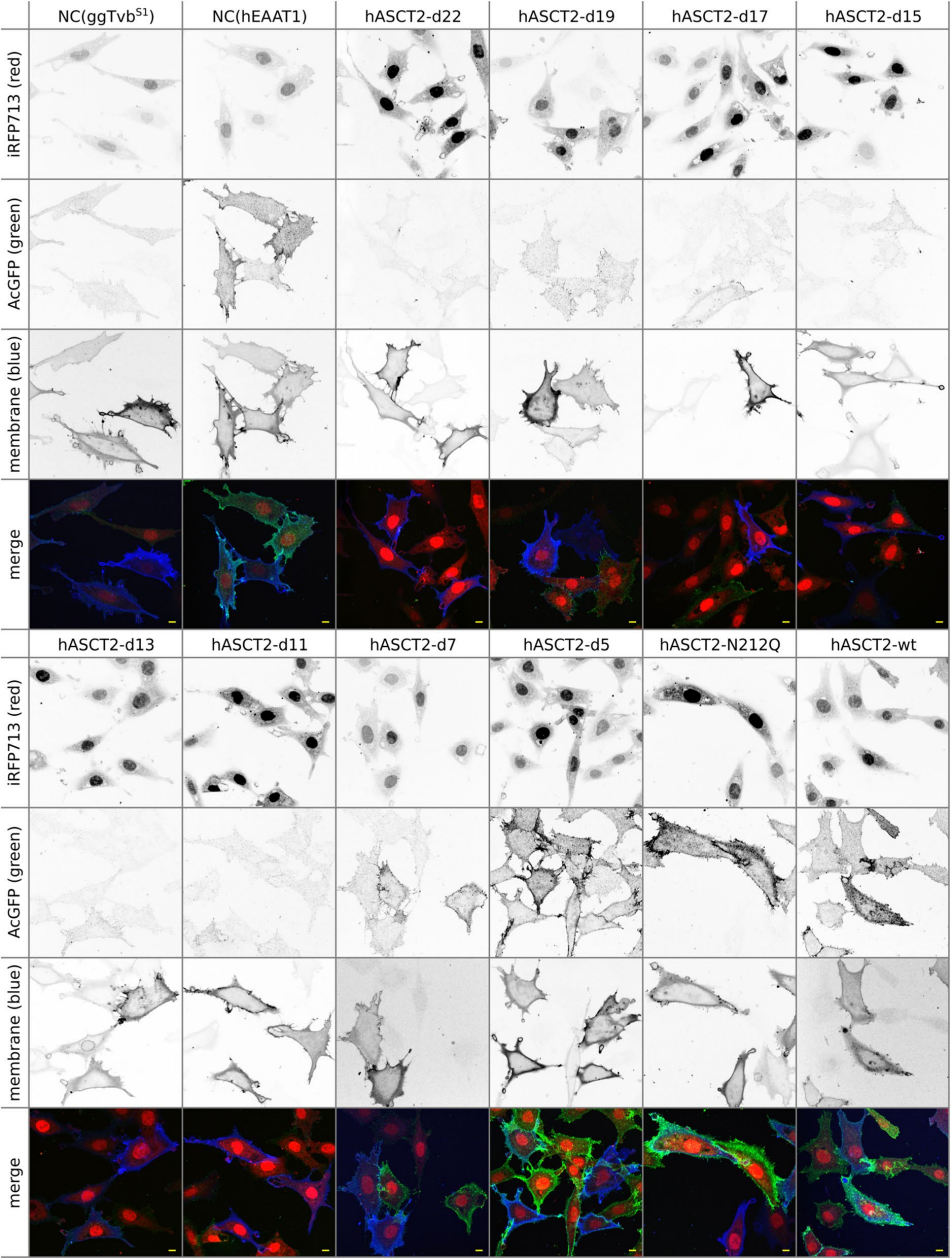
Cellular localisation of protein products of FuTraP variants. Confocal microscopy analysis showing the channels separately in grayscale and together in colourised composite. From top to bottom: the IRES-driven iRFP713 (red channel); AcGFP fused with ASCT2 (green channel); the stained cellular membrane (blue channel); merge of previous channels. Scale bars represent 10 μm. NC, negative control (ggTvb^S1^ or hEAAT1)

The correct display of the ASCT2 protein on the cell surface is crucial for its receptor function. To track ASCT2 localisation, we used confocal microscopy of ASCT2 mutants. Wild-type human ASCT2 localised preferentially to the cell surface, which was visualised after fluorescent staining of the cellular membrane (Fig. 2, bottom-right). Similarly, both the FuTraP-hASCT2-d5 deletion mutant and the FuTraP-hASCT2-N212Q glycosylation mutant preferentially localised to the cell surface (Fig. 2, green and blue channel). Corresponding to the FACS results, the rest of ASCT2 deletion mutants displayed a sharp reduction in the ASCT2 protein amount, while their cellular localisation could not be determined (Fig. 2). In contrast to AcGFP, iRFP713 was distributed throughout the entire cell, including the nucleus, in all FuTraP variants (Fig. 2, red channel). Moreover, we verified the localisation of FuTraP-ASCT2 variants using the Western blotting of surface proteins visualised by means of anti-ASCT2 antibody. The results confirmed the correct localisation of FuTraP-hASCT2-wt and showed reduced amounts of ASCT2 mutants with increasing deletion of region C on the cell surface (Additional file 1: Fig. S1c).

**Fig. 3:**
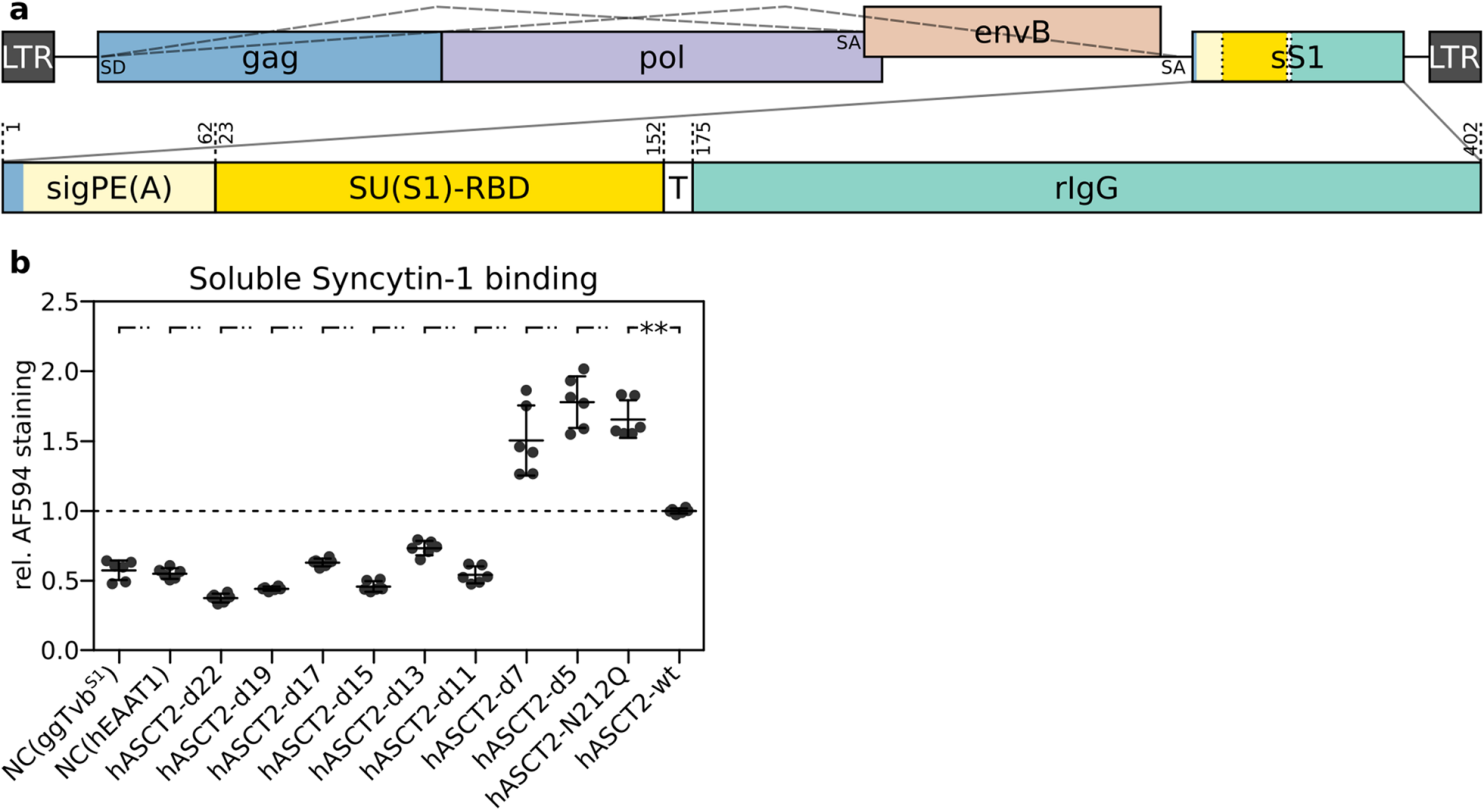
Binding of soluble Syncytin-1 to FuTraP-ASCT2 variants expressed on the cell surface. **a** Schematic representation of the vector expressing the soluble form of Syncytin-1 (sS1). sS1 was cloned downstream of the second splice acceptor (SA) of replication-competent avian retrovirus RCASBP(B), which permits propagation in cell culture and shedding of sS1 into the supernatant. sS1 consists of the signal peptide of Env(A) (sigPE(A)) followed by the in-frame receptor-binding domain of Syncytin-1 (SU(S1)-RBD), Tobacco Etch Virus protease cleavage site (T), and rabbit heavy chain of IgG (rIgG). sigPE(A) is composed of six amino acids from the retroviral Gag coding sequence (blue) followed by 56 amino acids from Env(A) (light yellow). The numbering corresponds to the original protein sequences. Long dashed lines illustrate two possible spliced products using the same splice donor (SD) and two alternative SA. The sS1 protein is produced to the supernatant (together with RCASBP(B) virus) and used for the cell surface labelling of ASCT2 variants and for inhibition experiments (Figs. 4 and 5). **b** Labelling of living cells with sS1. DF-1/LgBiT cells expressing either variants of FuTraP were incubated with sS1, and its binding was visualised by staining with anti-rabbit IgG antibody conjugated to Alexa Fluor 594. Median of Alexa Fluor 594 fluorescence intensity was measured by flow cytometry for individual FuTraP variants and normalised to the wild-type FuTraP-hASCT2-wt (Y-axis). LgBiT cells expressing the S1 allele of chicken Tvb (ggTvb^S1^) or human EAAT1 (hEAAT1) represent the negative control (NC). Results of three independent experiments performed in duplicates are shown. Data is shown as means ± standard errors, **p < 0.01

To further examine the ASCT2 display and its binding to Syncytin-1, we adapted immunoadhesin, a soluble fusion protein that combines the target-binding region of a ligand with the Fc region of an IgG [31–33]. We collected the supernatant containing the soluble extracellular form of Syncytin-1 (sS1) consisting of Syncytin-1 RBD fused with the heavy chain of rabbit IgG (Fig. 3a, Additional file 2: Fig. S2). Cells expressing ASCT2 variants were incubated with the supernatant containing sS1, and the binding to the ASCT2 receptor on the cell surface was visualised by an Alexa Fluor 594-conjugated anti-IgG antibody. FuTraP-hEAAT1 and FuTraP-ggTvb^S1^ cells labelled with sS1 served as negative controls. Flow cytometry analysis showed a specific shift in Alexa Fluor 594 staining, demonstrating the binding of sS1 to the wild-type FuTraP-hASCT2-wt (Fig. 3b). The FuTraP-hASCT2-d5, FuTraP-hASCT2-d7 deletion mutants and the FuTrap-hASCT2-N212Q glycosylation mutant bound sS1 even better than the wild-type. The results further showed decreased interaction of cells expressing the FuTraP-hASCT2-d11 to FuTraP-hASCT2-d22 deletion mutants with sS1 (Fig. 3b). The results confirmed the surface localisation of the ASCT2 receptor expressed from the pFuTraP vector, hence demonstrated the functional interaction of the receptor with the soluble form of Syncytin-1. These observations further supported the decreased surface display of ASCT2 mutants missing 11 or more amino acids of region C.

### ASCT2 receptor mediates entry of Syncytin-1-enveloped virus

**Fig. 4:**
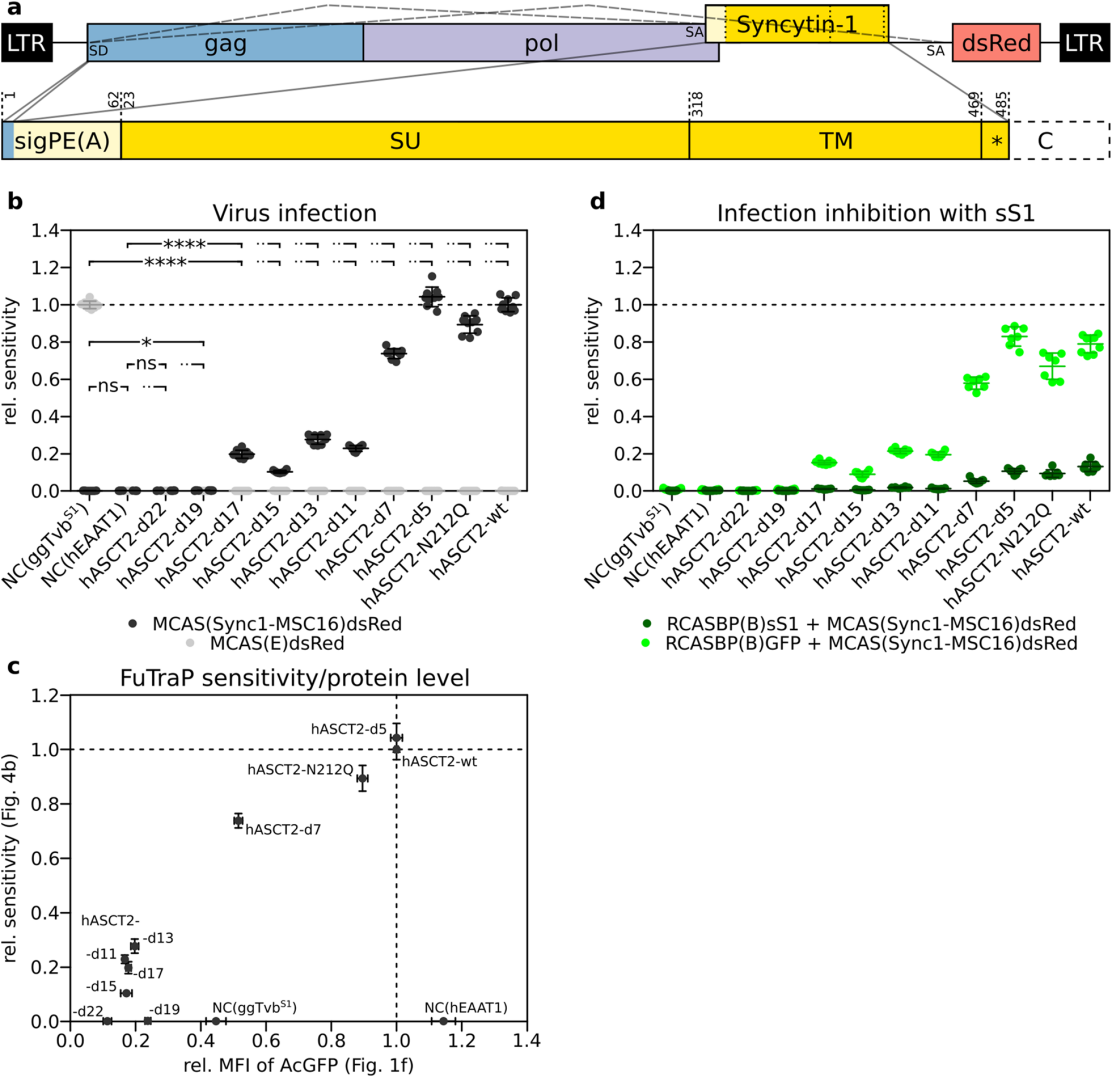
Virus infections of cells expressing the FuTraP-ASCT2 variants. **a** Scheme of the pMCAS(Sync1-MSC16)dsRed vector used for virus production. The envelope of the virus consisted of the signal peptide of Env(A) (sigPE(A)) followed in-frame by Syncytin-1 SU and TM. The first six amino acids before the splicing donor (SD) are shared by Gag and sigPE(A). The mutated cryptic splice acceptor of Syncytin-1 is depicted by an asterisk in the cytoplasmic tail (C), which was truncated to 16 amino acids. Dashed lines indicate alternative splicing generating Syncytin-1 mRNA and dsRed mRNA. Numbering corresponds to original protein sequences. **b** Infection of cells expressing variants of FuTraP with viruses enveloped by Syncytin-1 (black) or Env(E) (gray). Infected cells were identified by flow cytometry as dsRed-positive. Sensitivity to viral infection was normalised to the wild-type FuTraP-hASCT2-wt (Y-axis). Results of three independent experiments in biological triplicates are plotted. See Additional File 3: Fig. S3 for representative dot plots. **c** FuTraP sensitivity depends on the receptor expression. The data from **b** (Y-axis) were plotted against the ASCT2 protein level from Fig. 1f (X-axis). **d** Inhibition of infection by preincubation of FuTraP cells with sS1-containing supernatant (dark green). Preincubation with supernatant containing RCASBP(B)GFP was used as a mock control (light green). Following preincubation, the cells were infected with Syncytin-1-enveloped virus and fraction of dsRed-positive cells was analysed by flow cytometry. Sensitivity to viral infection was normalised to the wild-type FuTraP-hASCT2-wt (Y-axis). Results of three independent experiments in biological triplicates are plotted. DF-1/LgBiT cells expressing the S1 allele of chicken Tvb (ggTvb^S1^) or human EAAT1 (hEAAT1) represent the negative controls (NC). Data is shown as means ± standard errors, *p < 0.05, ****p < 0.0001, Mann–Whitney two-tailed test

To explore the ASCT2 capacity to mediate retroviral cellular entry, we adapted the human Syncytin-1 glycoprotein to act as a functional envelope protein of infectious avian retrovirus. We constructed chimeric replication-competent vector pMCAS(Sync1-MSC16)dsRed, in which the Syncytin-1 glycoprotein replaced the original envelope of ALV. After elimination of a cryptic splicing acceptor site and shortening the cytoplasmic domain of Syncytin-1 to 16 amino acids [10] (Fig. 4a), we obtained infectious MCAS(Sync1-MSC16)dsRed virus produced in the supernatant of transfected DF-1 cells. The virus reached a titre of 10^4^ IU/ml and was used to infect cells with multiplicity of infection 0.1. To obtain an irrelevant envelope protein as a negative control, we constructed the pMCAS(E)dsRed vector containing the envelope glycoprotein of ALV subgroup E. Infectious virus MCAS(E)dsRed was able to infect only the FuTraP-ggTvb^S1^-expressing cells because the DF-1 cells normally encode subgroup E-resistant receptor variant Tvb^S3^ (Fig. 4b, Additional file 3: Fig. S3).

The Syncytin-1-enveloped virus specifically entered cells expressing the wild-type FuTraP-hASCT2-wt, as we identified by dsRed fluorescence microscopically or by flow cytometry (6.9% dsRed-positive cells, Additional file 3: Fig. S3 bottom-right). Based on the percentage of dsRed-positive cells, we calculated the cellular sensitivity to viral infection considering the probability of simultaneous co-infections of the same cell (Fig. 4b). Importantly, no viral infection of FuTraP-hEAAT1 and FuTraP-ggTvb^S1^ cells (negative controls) by Syncytin-1-enveloped virus was detected (Fig. 4b, Additional file 3: Fig. S3). It is of note that the infectivity of the replication-competent retrovirus with Syncytin-1 envelope validates the supposed original function of Syncytin-1 as an envelope of ancestral exogenous retrovirus.

To determine whether deletion mutants can serve as receptors for the Syncytin-1-enveloped virus, we infected DF-1/LgBiT cells carrying ASCT2 variants with the MCAS(Sync1-MSC16)dsRed virus. The cellular sensitivity to infection was normalised to that of the FuTraP-hASCT2-wt cells and plotted as relative sensitivity. Both deletion mutant FuTraP-hASCT2-d5 and glycosylation mutant FuTraP-hASCT2-N212Q conferred similar sensitivity to infection as the ASCT2 wild-type FuTraP-hASCT2-wt (Fig. 4b). On the other hand, cells carrying FuTraP-hASCT2-d7 to FuTraP-hASCT2-d17 displayed reduced sensitivity to infection compared to cells carrying the wild-type FuTraP-hASCT2-wt (Fig. 4b). Nevertheless, in all these mutants, populations of dsRed-positive cells could be clearly identified after FACS analysis, including 1.7% of dsRed-positive FuTraP-hASCT2-d17 cells (Additional file 3: Fig. S3). FuTraP-hASCT2-d19 and FuTraP-hASCT2-d22 cells were resistant to infection (Fig. 4b, Additional file 3: Fig. S3).

Because the sensitivity of cells modified with the ASCT2 variant may depend on the amount of the receptor, we correlated the cellular sensitivity to infection as calculated from the fraction of dsRed-positive cells to the receptor protein level (AcGFP MFI of non-infected cells) (Fig. 4c). Diagonal distribution of the protein levels of receptor variants indicates the dependence of ASCT2 sensitivity on the extent of region C deletion in FuTraP-hASCT2 variants (Fig. 4c).

To verify the specificity of infection, we used sS1 to inhibit entry of the Syncytin-1-enveloped virus. Preincubation with sS1 reduced the infection sensitivity of all FuTraP-hASCT2 variants (Fig. 4d). In the case of FuTraP-hASCT2-d11 to FuTraP-hASCT2-d17 mutants, the sS1 treatment abolished infection with the Syncytin-1-enveloped virus, thus confirming that the detected low-level sensitivity conferred by these mutants represented specific infection (Fig. 4d).

To enhance viral infection and distinguish low levels of viral sensitivity from the background, we employed spinoculation of FuTraP variants with MCAS(Sync1-MSC16)dsRed and MCAS(E)dsRed viruses. Using spinoculation, the Syncytin-1-enveloped virus infected 56% of FuTraP-hASCT2-wt and 21% of FuTraP-hASCT2-d17 cells, further confirming the sensitivity of hASCT2 mutant missing 17 amino acids of region C (Additional file 4: Fig. S4). FuTraP-hASCT2-d19 and FuTraP-hASCT2-d22 mutants remained resistant after spinoculation. The relative sensitivity of mutants to spinoculated Syncytin-1-enveloped virus remarkably resembled the relative sensitivity after classical infection (compare Fig. 4b and Additional file 4: Fig. S4). Our results demonstrate that major deletion of the putative retrovirus docking site did not abolish the ASCT2 receptor interaction with the Syncytin-1 envelope and points to the importance of amino acids lying at the base of the protruding region C loop.

### Cell–cell fusion triggered by Syncytin-1

**Fig. 5:**
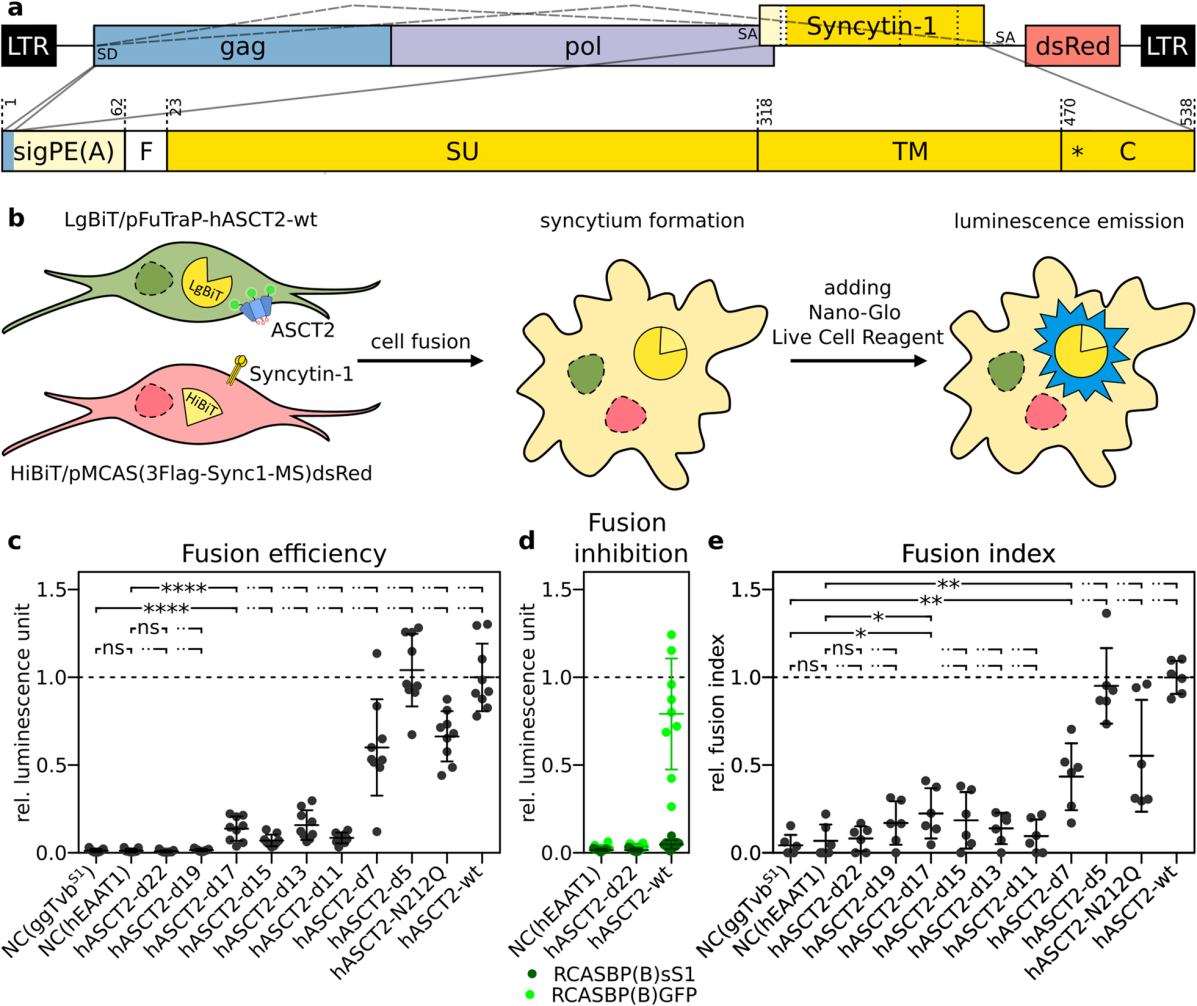
Cell–cell fusion luciferase assay. **a** Scheme of the pMCAS(3Flag-Sync1-MS)dsRed construct used for luciferase assay. The signal peptide of Env(A) (sigPE(A)) was followed in-frame by the three-Flag epitope (F), Syncytin-1 SU and TM subunits. The first six amino acids before the splicing donor (SD) are shared by Gag and sigPE(A). The construct contained the full-length cytoplasmic tail of Syncytin-1 (C). The mutated cryptic splice acceptor is depicted by an asterisk, dashed lines indicate the alternative splicing generating Syncytin-1 and dsRed mRNAs. Numbering corresponds to original protein sequences. **b** Scheme of the luciferase assay using the NanoBiT technology. Two subunits of NanoLuc luciferase, LgBiT and HiBiT, were transfected into a DF-1 cell line separately and stable transfectants were selected. Cells expressing the LgBiT part of NanoLuc luciferase were transduced with FuTraP variants and sorted (green). Cells expressing HiBiT were transiently transfected by pMCAS(3Flag-Sync1-MS)dsRed (red). A mixture of LgBiT- and HiBiT-expressing cells was seeded. The interaction of human ASCT2 with Syncytin-1 triggered cell–cell fusion followed by complementation of LgBiT by HiBiT. After substrate addition, the luminescence emission signal was quantified. **c** Efficiency of cell–cell fusion induced by interaction of Syncytin-1 with cells expressing variants of FuTraP measured by luciferase assay. Luminescence units of individual FuTraP variants were normalised to the wild-type FuTraP-hASCT2-wt (Y-axis). The experiment was repeated three times in biological triplicates. **d** Inhibition of fusion by preincubation of selected FuTraP cells with sS1-containing supernatant (dark green). Supernatant containing the RCASBP(B)GFP was used as a mock control (light green). Fusions were detected by the luciferase assay and luminescence units were normalised to non-treated wild-type FuTraP-hASCT2 (Y-axis). The experiment was repeated three times in biological triplicates. **e** Efficiency of cell–cell fusion induced by interaction of Syncytin-1 with cells expressing variants of FuTraP measured as a fusion index. The fusion index was defined as S/N, where S is the total number of nuclei in syncytia (≥ 3 nuclei within iRFP and dsRed double-positive cells) and N is the total number of nuclei in the field. The fusion index was normalised to FuTraP-hASCT2-wt (Y-axis). The experiment was repeated twice in biological triplicates. DF-1/LgBiT cells expressing the S1 allele of chicken Tvb (ggTvb^S1^) or human EAAT1 (hEAAT1) represent the negative controls (NC). Data is shown as means ± standard errors, *p < 0.05, **p < 0.01, ****p < 0.0001 Mann–Whitney two-tailed test

Finally, we focused on the cell–cell fusion triggered by the ASCT2-Syncytin-1 interaction. We constructed non-infectious retroviral expression vector pMCAS(3Flag-Sync1-MS)dsRed. In this construct, the original ALV retroviral envelope was replaced by the entire Syncytin-1 open reading frame with an N-terminally fused three-Flag epitope and mutated cryptic splice acceptor (Fig. 5a). After transfection of ASCT2-expressing cells with pMCAS(3Flag-Sync1-MS)dsRed, only a negligible amount of infectious viral particles was produced (Additional file 5: Fig. S5), but importantly, we observed cell–cell fusion, which further supported the correct ASCT2 surface localisation and receptor function (Additional file 6: Fig. S6, Additonal file 7: Movie).

To quantify cell–cell fusion, we utilised the NanoLuc Binary Technology (NanoBiT), where the High-Affinity NanoBiT (HiBiT) subunit spontaneously complements the Large NanoBiT (LgBiT) subunit to form a functional NanoLuc luciferase enzyme (Fig. 5b). In addition to DF-1/LgBiT cells with FuTraP variants described above, we engineered DF-1 cells that stably expressed HiBiT and modified them to express fusogenic Syncytin-1. The pMCAS(3Flag-Sync1-MS)dsRed vector was transfected into DF-1/HiBiT cells, and a transfection efficiency of 36% was determined according to dsRed fluorescence (Additional File 8: Fig. S7). Anti-Flag cell labelling showed that 42% of the transfected cells expressed Syncytin-1 on the cell surface (Additional file 8: Fig. S7).

Finally, DF-1/LgBiT cells expressing FuTraP variants were seeded together with DF-1/HiBiT cells that had been transfected with Syncytin-1, and cell–cell fusion was quantified as NanoLuc luciferase luminescence (Fig. 5c). DF-1/LgBiT/FuTraP-hEAAT1 and DF-1/LgBiT/ggTva^S1^ cells mixed with DF-1/HiBiT-Syncytin-1 cells were used as negative controls. Our results revealed the fusion ability of FuTraP-hASCT2-wt, FuTraP-hASCT2-N212Q, and FuTraP-hASCT2-d5 to FuTraP-hASCT2-d17 deletion mutants that was higher than that of negative controls (Fig. 5c). Increasing the extent of deletion within region C led to a decrease in the fusion ability. FuTraP-hASCT2-d19 and FuTraP-hASCT2-d22 mutants did not support cell–cell fusion (Fig. 5c). The observed cell–cell fusion was specifically triggered by Syncytin-1, because sS1 inhibited the fusion activity of FuTraP-hASCT2-wt (Fig. 5d). Furthermore, the fusion index determined microscopically as the fraction of syncytialized nuclei was in accordance with the quantitative luciferase assay (Fig. 5e). Our results further confirmed that the interaction of Syncytin-1 with ASCT2 expressed from the dual-reporter FuTraP system triggered cell–cell fusion. The luciferase fusion assay corroborated our conclusions that progressive deletions of region C reduced the ASCT2 cell surface expression. We further demonstrated that the mutant with removed 17 amino acids of region C was able to interact with the Syncytin-1 envelope glycoprotein.

## Discussion

We have created a heterologous retroviral system, FuTraP, for precise analysis of the ASCT2 protein amount and cell membrane localisation. Our system allows quantitative assessment of the ASCT2-Syncytin-1 interaction at multiple levels, including Syncytin-1 binding, membrane fusion, and cellular entry of the Syncytin-1-enveloped virus. Our report demonstrates another advantage of the presented system, which consists of accessible large-scale screening of ASCT2 mutants. The presented system can be easily modified for analyses of the interactions between different RDR Envs and ASCT2 or ASCT1 receptors. Additionally, we propose that the FuTraP system could be suitable for expression of other transmembrane proteins and analysis of their receptor interactions.

We suggest that the FuTraP system will be useful for further studies of ASCT2 interaction. The precise site of interaction with Syncytin-1 has not been identified yet, despite extensive analyses performed using human, mouse, and hamster ASCT2 chimaeras and glycosylation mutants [1, 12]. The findings of previous studies pointed to the role of region C [24], which can be interpreted either as the viral attachment site or the negative control region inhibiting access of Syncytin-1 to an unidentified interaction site. We tried to resolve the question, but our initial experiments were limited by inefficient expression and mislocalisation of ASCT2 variants bearing deletions of region C. This led us to development of the FuTraP system.

The FuTraP system allows multi-level assessment in a single cytometry experiment. The dual-fluorescence arrangement enables us to easily distinguish between reduced Syncytin-1-receptor affinity due to modification of the interaction site, or decreased ASCT2 protein amount. Our transduction-based system that presumably results in precise integration of a single-copy retroviral vector has proved to be essential in the analysis of ASCT2 region C deletion mutants. The measurement of iRFP713 fluorescence validated a similar mRNA amount for all deletion variants, while AcGFP fluorescence clearly demonstrated that progressive deletion of the protruding region C substantially decreased the amount of ASCT2 protein within the cells.

Importantly, our experiments demonstrated that DF-1 cells without ectopic expression of human ASCT2 were not sensitive to Syncytin-1-enveloped virus at all, underlining the benefits of the heterologous chicken system. The zero background means that endogenous chicken ASCT1 is not compatible with Syncytin-1. Thus, the surrogate human ASCT1 or ASCT2 expression in our system can help in understanding of individual contribution of ASCT1 and ASCT2 to the fusogenic capacity of human trophoblast. It is not clear whether both alternative receptors have equal and additive effects or whether one of them is the major player in the cell–cell fusion process.

As an additional tool to explore the interaction of ASCT2 variants with Syncytin-1, we adapted an immunoadhesin assay based on the soluble fusion protein sS1 combining the receptor-binding domain of Syncytin-1 with the Fc region of an IgG. Specifically, we employed the immunoadhesin to assess the cell surface localisation of ectopically expressed ASCT2 mutants and their binding to Syncytin-1 RBD. The method was less sensitive than the tests of virus infectivity or the fusion assay and was substantially dependent on the level of receptor expression on the cell surface. Although the binding mechanism of homodimeric sS1-RBD-immunoadhesin may not exactly reflect that of the native trimeric SU-TM subunits of Syncytin-1, we observed specific association of sS1 with the wild-type ASCT2 receptor (Fig. 3b). Furthermore, we anticipate that this association explains the sS1-specific inhibition of Syncytin-1-enveloped virus infection and Syncytin-1-triggered cell–cell fusion (Figs. 4d, 5d). The results of our immunoadhesin assay are in accordance with virus infection and cell–cell fusion experiments and imply that the distal part of region C is not responsible for the interaction with Syncytin-1.

Next, we modified Syncytin-1 to function as the envelope of a chimeric infectious avian retrovirus. Although Syncytin-1 has been shown to pseudotype lentiviral particles [12, 24], our results demonstrate successful recovery of the endogenous Syncytin-1 glycoprotein as an envelope of a replication-competent retrovirus. We have shown that in addition to promoting cell–cell fusion, Syncytin-1 can perform its expected original function, i.e., mediate infection of cells that express the specific receptor.

Based on the cellular sensitivity, we suggest that ASCT2 was susceptible to Syncytin-1 infection after deletion of 17 amino acids from region C. We detected a sharp decline in receptor sensitivity when comparing FuTraP-hASCT2-d17 to FuTraP-hASCT2-d19 mutant (Fig. 4b, c). This result was further supported by spinoculation (Additional file 4: Fig. S4) and confirms that the cellular entry of the Syncytin-1-enveloped virus would be the most sensitive assay to disclose functional Syncytin-1-receptor interaction, especially under the conditions of the reduced protein level of receptor variants.

Interestingly, FuTraP-hASCT2-d19 did not interact with Syncytin-1 despite the fact that it expressed a relatively high amount of ASCT2 protein that was localised on the cell surface (Fig. 1f, g, Fig. 4c, Additional file 1: Fig. S1). On the other hand, our data concerning the interaction of FuTraP-hASCT2-d22 with Syncytin-1 are inconclusive because of the very low level of protein expression and unknown localisation. According to the cryo-EM model and structure predictions, region C is composed of a beta-sheet-resembling stretch of amino acids that protrudes towards the extracellular space, a turn with N212 amino acid making the tip of region C, and a second beta-sheet-resembling stretch coming back to the receptor bowl-shaped core [25, 26]. Region C is presumably engaged in Syncytin-1 binding; however, its precise role remains unclear. It could contain the interaction site, it could participate as one of the several co-operative sites of Syncytin-1 binding, or it could inhibit the Syncytin-1 access to its interaction site(s). We have demonstrated that the most protruding portion of region C is not the direct interaction site. Conclusions concerning the FuTraP-hASCT2-d19 resistance vs FuTraP-hASCT2-d17 sensitivity should be considered cautiously, because by deleting a large part of region C we could have changed the local fold of the receptor core and could have artificially exposed or masked the amino acids important for Syncytin-1 interaction. We suggest that we have reached the limitation of deletion analysis that, in particular, has demonstrated the importance of amino acids located at the bases of antenna-protruding region C. At this point, substitution mutants in the FuTraP context would be more suitable to resolve the ASCT2-Syncytin-1 interaction. In summary, the results of the virus infection assay indicate that the distal 17 amino acids of region C do not represent the interaction site with the Syncytin-1 glycoprotein.

The ECL2 region of ASCT2 contains two glycosylated asparagine residues—N163 and N212. Creation of a double mutant in which both glycosylations were eliminated was shown to alter the ASCT2 protein level and localisation [27]. We mutated the N212 glycosylation site within region C and assessed the single glycosylation mutant for sensitivity to Syncytin-1-triggered infection and cell–cell fusion. Importantly, we confirmed that the FuTraP-N212Q mutant was correctly expressed and localised on the surface of avian DF-1 cells. Our results showed that receptor N212 glycosylation contributed to proper expression, but was not essential for interaction with Syncytin-1.

Finally, we explored the efficiency of cell–cell fusion induced after Syncytin-1 interaction with the ASCT2 variants. Cell–cell fusion is routinely detected by May-Grünwald and Giemsa staining followed by microscopic techniques, optionally using indicator cells expressing β-galactosidase or GFP [1, 2, 12, 24, 34, 35]. In this case, quantitation is achieved by nuclei and syncytia counting and/or by colorimetric and fluorescent assays. Recently, complementation of a reporter protein, either GFP or luciferase, was introduced to quantify cell–cell fusion [36–38]. To quantitatively assess Syncytin-1-triggered cell–cell fusion, we adapted the NanoLuc Binary Technology complementation assay. We selected this technology because it uses NanoLuc luciferase, which produces high intensity, glow-type luminescence after spontaneous assembly of High-Affinity NanoBiT and Large NanoBiT subunits. These parameters provided high-sensitivity quantitative measurement of the cell–cell fusion. Results of the luciferase quantitative assay were validated by microscopic detection of cell–cell fusion. The assay confirmed differences in the fusion ability among the ASCT2 mutants. Similarly to the assessment of virus infection, we detected a functional interaction between Syncytin-1 and all ASCT2 variants except for FuTraP-hASCT2-d19 and FuTraP-hASCT2-d22 mutants. The cell–cell fusion results correspond to viral infectivity conclusions and bring attention to the amino acids located at the bases of region C and their contribution to Syncytin-1 interaction. Our results imply that while region C is essential for the receptor surface expression, its protruding distal part is not directly responsible for the interaction with Syncytin-1.

## Conclusions

We introduce FuTraP, a novel system to study the expression and interaction of transmembrane proteins based on fluorescent and luminescent techniques. We employed FuTraP for heterologous ectopic expression of the human ASCT2 receptor in chicken cells. We have demonstrated the benefits of FuTraP on a panel of ASCT2 mutants containing deletions within the region C of extracellular loop 2. We have shown that deletion of a major part of region C affected the ASCT2 protein level. Region C was earlier proposed as the part of the receptor that is crucial for the docking of several retroviruses. We focused on the interaction of ASCT2 with Syncytin-1, an envelope of human endogenous retrovirus. To evaluate the interaction of ASCT2 mutants with Syncytin-1, we developed sensitive assays that measured soluble Syncytin-1 binding, sensitivity to infectious virus and cell–cell fusion. We have demonstrated that glycosylation of region C is not required for the interaction of Syncytin-1 and ASCT2. Further, our results show that deletions of the protruding distal part of region C do not abolish the receptor function. Our system can facilitate precise characterisation of the Syncytin-1 binding site on the receptor and lead to detailed molecular understanding of one of the critical steps in human placenta morphogenesis.

## Methods

### Cloning of expression vectors and viruses

Sequences of all constructs used in the study are accessible in Additional files 9, 10, 11, 12, 13, 14, 15, 16, 17, 18, 19, 20, 21, 22, 23, 24, 25. Coding sequences of human EAAT1, ASCT2 and Syncytin-1 were amplified from the BeWo choriocarcinoma cell line cDNA. The coding sequence of chicken Tvb allele S1 was amplified from chicken L15 primary fibroblasts cDNA and cloned with a deletion of amino acids 276–363 where the C-terminal death domain was encoded. cDNA was synthesised by AccuScript polymerase (Agilent) with oligo(dT) primers. The sequences corresponded to GenBank NP_004163.3, NP_005619.1, NP_001124397.1 and NP_989446.2, respectively. For all cloning steps, an In-Fusion Cloning Kit (TaKaRa) was used.

The pFuTraP vector (Fig. 1a) was based on replication-deficient avian retroviral vector pRNIG used in our laboratory [39]. pFuTraP contained LTR sequences derived from Myeloblastosis Associated Virus (MAV, GenBank accession No. L10922.1, [40]) and the encapsidation signal from RCASBP [41]. Downstream of the encapsidation signal, a Kozak’s sequence, the coding sequences of human EAAT1, ASCT2 or chicken Tvb^S1^ were cloned. These membrane proteins possessed a mutated stop-codon, which allowed a read-through into GGGGS linker, and AcGFP fused in-frame. The IRES sequence derived from the encephalomyocarditis virus was placed downstream of AcGFP, which ensured translation of iRFP713, the far-red fluorescent protein. The vector was propagated in the Stbl2 strain of *E. coli*. pFuTraP containing the human EAAT1 was designated pFuTraP-hEAAT1 (Additional file 9), pFuTraP containing the chicken Tvb^S1^ was designated pFuTraP-Tvb^S1^ (Additional file 10), and the wild-type human ASCT2 was designated pFuTraP-hASCT2-wt (Additional file 11). pFuTraP-hASCT2 containing deletions that shortened the protruding antiparallel beta-sheets of region C by 5 to 22 amino acids were designated pFuTraP-hASCT2-d5 to pFuTraP-hASCT2-d22. Region C is composed of beta-sheets that protrude towards the extracellular space, turn at the tip and go back towards the cellular membrane. To favour the folding and compensate for the loop flexibility at the tip, we substituted the deleted amino acids by two glycines, flexible amino acids that would facilitate the bending of the tip of the loop. We cloned the following deletion mutants: pFuTraP-hASCT2-d5, pFuTrap-hASCT2-d7, pFuTraP-hASCT2-d11, pFuTraP-hASCT2-d13, pFuTraP-hASCT2-d15, pFuTraP-hASCT2-d17, pFuTraP-hASCT2-d19, pFuTraP-hASCT2-d22 (Fig. 1b, Additional files 12, 13, 14, 15, 16, 17, 18, 19). pFuTraP containing the ASCT2 N212Q glycosylation mutant was designated pFuTraP-hASCT2-N212Q (Fig. 1b, Additional file 20).

pMCAS(Sync1-MSC16)dsRed (Fig. 4a, Additional file 21) was a plasmid containing replication-competent avian retrovirus based on high-titre RCASBP [41, 42]. The LTR sequences were derived from MAV, the syncytin-1 coding sequence replaced the original ALV *env* gene, and the dsRed coding sequence was cloned downstream of the splice acceptor instead of the original v-*src* gene. The signal peptide of the *Syncytin-1* gene was replaced with the signal peptide of the ALV *env*(*A*) gene. The cryptic splice acceptor identified within the syncytin-1 coding sequence [43, 44] was mutated to increase the ratio of spliced syncytin-1 mRNA to spliced dsRed mRNA. To obtain infectious Syncytin-enveloped virions, a stop codon was introduced after 16 amino acids of the cytoplasmic tail as previously described [10] (Fig. 4a). After transfection of pMCAS(Sync1-MSC16)dsRed, infectious viral particles were produced. In contrast, pMCAS(Sync1-MS)dsRed and pMCAS(3Flag-Sync1-MS)dsRed (Fig. 5a, Additional files 22 and 23) expressing Syncytin-1 with the unshortened cytoplasmic tail did produce negligible amounts of infectious viral particles (Additional file 5: Fig. S5). In pMCAS(3Flag-Sync1-MS)dsRed vector, Syncytin-1 was fused N-terminally with the three-Flag epitope (Fig. 5a). All vectors were manipulated at the BSL2 containment level. pMCAS(E)dsRed (Additional file 24) was constructed from pMCAS(Sync1-MSC16)dsRed by replacement of the *syncytin*-1 coding sequence with *env*(E) [45].

### Cell lines, transfections, and transductions

In the experiments, chicken fibroblast cell line DF-1 [46], Avipack packaging cell line [47] and chicken L15 primary fibroblasts were used. All the cells were cultured in DMEM:F12 media (Sigma), supplemented with 4% foetal bovine serum, 4% bovine serum, and 1% chicken serum in 5% CO_2_ atmosphere at 37 °C. One hundred μg of penicillin and 100 μg streptomycin per millilitre of media were added. For all transfections, Lipofectamine 3000 (Thermofisher) was used according to the manufacturer’s instructions on cells in the exponential phase of growth.

The DF-1 cell line was further modified to express fragments of NanoLuc luciferase (Nano-Glo® HiBiT system, Promega). We separately transfected linearized vectors containing two fragments of the luciferase enzyme—LgBiT and HiBiT (Promega)—and after two-week selection in the presence of Hygromycin B (0.2 mg/ml), stable cell lines expressing either LgBiT or HiBiT proteins were obtained.

To achieve stable ectopic expression, the FuTraP genome was transduced into target DF-1/LgBiT cells by infection with VSV-G-pseudotyped viral particles. Transducing viruses were produced in the Avipack cell line after co-transfection of a 35-mm dish with 0.5 μg pVSV-G (Clontech), 0.75 μg pcgag-pol [39], and 1.25 μg pFuTraP. The supernatant containing the transducing virus was collected three days after transfection, filtered through a 0.45 μm filter, and applied to a 35-mm dish with 0.15 × 10^6^ DF-1 cells. Modified cells were cultivated for one week followed by sorting of the iRFP713-positive cell population. The transduction efficiency 2–10% led to 3–15 × 10^3^ integrations. The sorted cells were expanded for another week and sorting of the iRFP713-positive population was repeated. The resulting cell lines are designated FuTraP.

### Microscopy

The cells were seeded on microscope cover glasses (0.15 × 10^6^ cells per 35 mm dish with the cover glass) and incubated overnight. The next day, the cells were fixed in 4% paraformaldehyde, washed with PBS, stained with membrane dye CellBrite Blue (Biotium) according to the manufacturer’s protocol, mounted in PBS, and visualised using Andor Dragonfly 503 confocal spinning disc microscope with a 63 × /1.2 NA objective.

Images were deconvolved by Huygens software and contrast was enhanced using ImageJ. The signals of AcGFP and iRFP713 were adjusted uniformly over all mutants to illustrate the intensity of expression.

### Soluble Syncytin-1-immunoadhesin binding

Construction of the soluble form of Syncytin-1 pSU(S1)-RBD-IgG (Fig. 3a, Additional file 25) was based on immunoadhesin previously used in ALV receptor studies [32, 33]. pSU(S1)-RBD-IgG contained signal peptide of ALV *env*(*A*) followed in-frame by Syncytin-1 amino acids 23 to 152 of the SU subunit containing the putative RBD [11]. By means of a nine amino acid linker (Tobacco Etch Virus protease recognition sequence), the specified RBD of Syncytin-1 was fused to amino acids 175 to 402 of the constant region of the rabbit immunoglobulin G gene (GenBank Accession No. K00752.1). The resulting gene was inserted into replication-competent avian retroviral vector pRCASBP(B) downstream to the ALV env(B) gene and the second splice acceptor site (Fig. 3a). After transfection of pSU(S1)-RBD-IgG into the DF-1 cells, the replication-competent virus with the ALV envelope (subgroup B) was spread throughout permissive cell culture and, in addition, the infected cells produced the soluble form of Syncytin-1 (sS1) in the supernatant. After three passages of transfected cells, the supernatant that contained both sS1-immunoadhesin and RCASBP(B) virus was collected, filtered through a 0.45 μm filter, aliquoted, and stored at − 80 °C.

To measure the binding of sS1, the FuTraP variants were detached using a non-enzymatic cell dissociation solution (Sigma), and 2.5 × 10^5^ cells were incubated with 250 μl of supernatant containing sS1 at 4 °C for 1 h. The cells were washed three times with PBS supplemented with 2% bovine serum. Then, the cells were incubated for 30 min at 4 °C with anti-rabbit IgG conjugated to Alexa Fluor 594 antibody (1:1000 dilution in PBS with 2% bovine serum). After staining, the cells were washed three times in PBS supplemented with 2% bovine serum. Flow cytometry analysis was performed and the median fluorescence intensity of Alexa Fluor 594 was determined.

### Infections

Infectious virus MCAS(Sync1-MSC16)dsRed was produced by transfection of DF-1 cells; the supernatant was collected and filtered 2–3 days after the transfection. Infectious virus MCAS(E)dsRed was produced by transfection of sensitive chicken L15 cells; the supernatant was collected and filtered after three passages of transfected cells from a fully infected culture. The virus was titrated on FuTraP-ggTvb^S1^ cells and diluted by fresh medium to achieve the MOI comparable with MCAS(Sync1-MSC16)dsRed.

Infections were performed as follows: 5 × 10^4^ of pFuTraP cells were seeded one day before infection in a 24-well plate; cells were infected with 500 μl of virus supernatant; the supernatant was replaced with fresh media one day post-infection. Three days post-infection, the cells were fixed in 1–2% paraformaldehyde (final concentration), analysed by flow cytometry, and a fraction of infected cells was detected by dsRed fluorescence (Additional file 3: Fig. S3).

The sensitivity of cells was calculated using the following formula: $sensitivity\, = \,\, - \,{\text{ln }}\left( {{\text{1}}\, - \,fraction{\text{ }}of{\text{ }}dsRed{\text{ }}positive{\text{ }}cells} \right)$ [48]. The sensitivity to MCAS(Sync1-MSC16)dsRed and MCAS(E)dsRed was further normalised to the sensitivity of FuTraP-hASCT2-wt and FuTraP-ggTvb^S1^, respectively.

To assess the inhibition of infection by sS1, 5 × 10^4^ of FuTraP cells were seeded in 24-well plate. The next day, cells were preincubated with 100 μl of supernatant containing sS1 (as well as RCASBP(B)) or RCASBP(B)GFP alone. After 2 h at 37 °C, 400 μl of supernatant containing MCAS(Sync1-MSC16) was added. One day post infection, the medium was exchanged, and three days post infection, the cells were fixed and analysed by flow cytometry.

### NanoBiT Luciferase-based live-cell assay for cell–cell fusion quantification

To quantify cell–cell fusion, we adapted the NanoBiT technology based on two-subunit NanoLuc luciferase (Nano-Glo® HiBiT system, Promega). DF-1/HiBiT cells were seeded in a 6-well plate (0.45 × 10^6^ cells/well) and after 24 h transfected with 2.5 µg of pMCAS(3Flag-Sync1-MS)dsRed. Forty-eight hours after transfection, the DF-1/HiBiT-Sync1 cells were mixed with DF-1/LgBiT/FuTraP cells in ratios 2 × 10^4^: 1 × 10^4^ cells, respectively, and transferred in triplicate to a whole-white 96-well plate (Costar). For inhibition of cell–cell fusion, the mixture of DF-1/HiBiT-Sync1 and DF-1/LgBiT/pFuTraP cells was seeded in a whole-white 96-well plate and cultured in the medium containing either sS1 (as well as RCASBP(B)) or RCASBP(B)GFP alone. After 24 h incubation, the supernatant from the cells was replaced with 100 µl of OptiMEM medium and the cells were incubated for additional 60 min. Afterwards, 20 µl of 37 °C-equilibrated Nano-Glo Live Cell Reagent (19 µl of LCS Dilution Buffer and 1 µl of Live Cell Substrate; Promega) containing the luciferase substrate, furimazine, was added to cells and the plate was placed on an orbital shaker at 300 rpm, 15 s. At this point, reassorted HiBiT-LgBiT fragments in the fused cells started to oxidise the substrate, resulting in luminescence emission. The relative luminescence was measured in an EnVision Plate Reader (PerkinElmer) immediately after adding the Live Cell Reagent.

To verify the surface expression of 3Flag-Sync1-MS, 2.5 × 10^5^ living cells were immunostained 48 h after the transfection with monoclonal Anti-Flag® M2-FITC antibody (Sigma, 1:1000 dilution in PBS with 2% calf serum; 1 ml per 10^6^ cells) and evaluated by flow cytometry. A live gate was created according to Hoechst 33258 staining. Transfection efficiency was assessed by flow cytometry according to the dsRed fluorescence (Additional file 8: Fig. S7).

### Evaluation of cell–cell fusion by fluorescent microscopy

In parallel with Luciferase-based live-cell assay, we quantified cell–cell fusion using fluorescent microscopy and subsequent image analysis. Forty-eight hours after transfection, the DF-1/HiBiT-Sync1 cells were mixed with DF-1/LgBiT-pFuTraP cells in ratios 2 × 10^4^: 1 × 10^4^ cells, respectively, and transferred in triplicate to a 48-well plate. After 24 h incubation, the medium was replaced with 100 μl of FluoroBrite DMEM (Thermofisher) media with Hoechst 33342. Cells were incubated for 30 min and visualised using a Leica DMI8 microscope with a 20 × /0.40 NA objective. For analysis, nine randomly selected microscopy images of each sample triplicate (three fields per sample) were processed and analysed using ImageJ software. The fusion index, defined as S/N, where S is the total number of nuclei in syncytia (three or more nuclei within iRFP713 and dsRed double-positive cells) and N is the total number of nuclei in the field, was calculated for each image.

### Statistical analysis

For statistical analysis of the intergroup specificity, GraphPad Prism software (version 5.04) was employed. Non-parametric two-tailed Kruskal–Wallis test was followed by Mann–Whitney test. Calculated P-values of Mann–Whitney test were depicted as follows: **** < 0.0001, *** < 0.001, ** < 0.01, * < 0.05, ns > 0.05.

## Supplementary Information

**Additional file 1: Fig. S1.** Western blot analyses of FuTraP-ASCT2 variants.** a** Western blot analysis of the FuTraP cell lysates using anti-ASCT2 antibody. Cells were lysed in a solution containing 0.1 M Tris pH 7.6, 7 M urea, 1.5% Triton X-100, mixed with an SDS-containing sample buffer, subjected to 12% SDS-PAGE, and transferred onto PVDF membrane with a semidry system. ASCT2 was detected using a rabbit polyclonal anti-ASCT2 antibody (HPA035240, Sigma-Aldrich) diluted 1:700. Horseradish peroxidase-conjugated secondary goat anti-rabbit antibody (Cell Signaling) and SuperSignal West Femto (ThermoScientific) were used for chemiluminescence detection. The wild-type ASCT2-AcGFP protein has expected molecular weight 84 kDa. **b** Western blot loading control of the FuTraP cell lysates using anti-GAPDH antibody. The membrane from **a** was incubated with mouse monoclonal anti-GAPDH antibody (GA1R, ThermoFisher, dilution 1:3000) and detected as in **a**. **c** Western blot analysis of the FuTraP cell surface proteins using anti-ASCT2 antibody. Cell surface proteins were isolated using a Pierce Cell Surface Isolation Kit (Thermo Scientific) according to manufacturer’s instructions. Briefly, cell surface proteins on the living cells were biotinylated, isolated with NeutrAvidin Agarose™ and eluted in the SDS lysis buffer. Further analysis was performed as in **a**. DF-1/LgBiT cells expressing the S1 allele of chicken Tvb (ggTvb^S1^) or human EAAT1 (hEAAT1) represent the negative controls (NC). kDa molecular weights are indicated.

**Additional file 2: Fig. S2. **sS1-immunoadhesin produced to the supernatant. sS1-immunoadhesin containing the Fc region was immunoprecipitated from the collected supernatant by protein G-Agarose (Pierce, ThermoScientific), mixed with an SDS-containing sample buffer, subjected to 12% SDS-PAGE and transferred onto PVDF membrane with a semidry system. sS1-immunoadhesin was detected using horseradish peroxidase-conjugated goat anti-rabbit IgG antibody (Cell Signaling) and SuperSignal West Femto (ThermoScientific). Separately collected supernatants containing sS1 (as well as RCASBP(B)) that were used for labelling of FuTraP-expressing cells, for inhibition of infection or cell–cell fusion or for purification, were analysed. In contrast to sS1 in the supernatant, the purified sS1 (mAbTrap Kit, Cytiva) was not functional in cell labelling or inhibition experiments and was not used further. Mock control supernatant containing the RCASBP(B)GFP only was used as a negative control (NC).

**Additional file 3: Fig. S3. **Infection of cells expressing FuTraP-ASCT2 variants with Syncytin-1-enveloped virus. Cells modified with variants of FuTraP were infected with MCAS(Sync1-MSC16)dsRed or mock-infected with MCAS(E)dsRed and analysed by flow cytometry. Representative dot plots of 10^4^ cells are shown. The X-axis depicts the AcGFP fluorescence, Y-axis depicts the dsRed fluorescence. The fraction of infected (dsRed-positive) cells was gated. Negative control (NC) is represented by FuTraP-hEAAT1 or FuTraP-ggTva^S1^.

**Additional file 4: Fig. S4. **Spinoculation of cells expressing FuTraP-ASCT2 variants with Syncytin-1-enveloped virus. **a** Spinoculation of cells expressing variants of FuTraP with viruses enveloped by Syncytin-1 (black) or Env(E) (gray). Spinoculation was performed as follows: 25 × 10^3^ cells in a 48-well plates were centrifuged with 250 μl of virus supernatant in the presence of 8 μg/ml of polybrene, for 2 h at 1200 × *g* at 25 °C and the supernatant was replaced with fresh media after the centrifugation. Three days post-infection, the cells were fixed in 1–2% paraformaldehyde (final concentration), analysed by flow cytometry, and the fraction of infected cells was detected by dsRed fluorescence. Sensitivity to viral infection was normalised to the wild-type FuTraP-hASCT2-wt (Y-axis). Results of the representative experiment performed in biological duplicates are plotted. **b** Representative dot plots of Syncytin-1-enveloped virus spinoculation. FuTraP-hASCT2-wt, FuTraP-hASCT2-d17, FuTraP-hASCT2-d19 and FuTraP-hEAAT1 (NC) cells after spinoculation and flow cytometry analysis are depicted for illustration. The X-axis depicts the AcGFP fluorescence, Y-axis depicts the dsRed fluorescence. The fraction of infected (dsRed-positive) cells was gated. 10^4^ cells are shown.

**Additional file 5: Fig. S5.** Infectivity of Syncytin-1-enveloped viruses. **a** Transfection efficiency of three variants of Syncytin-1 plasmids. Seeded DF-1 cells were transfected by pMCAS(Sync1-MS)dsRed (red), pMCAS(3Flag-Sync1-MS)dsRed (green) or pMCAS(Sync1-MSC16)dsRed (blue). Two days post-transfection, the supernatant was collected and the cells were analysed by flow cytometry. X-axis depicts the dsRed fluorescence. **b** Reverse transcriptase activity produced to the supernatant by three variants of Syncytin-1. Two days post-transfection, the supernatant of the pMCAS(Sync1-MS)dsRed- (red), pMCAS(3Flag-Sync1-MS)dsRed- (green) or pMCAS(Sync1-MSC16)dsRed- (blue) transfected cells was analysed for production of reverse transcriptase activity to the supernatant by means of product-enhanced reverse transcriptase (PERT) assay (as published in Krchlikova V, Fabryova H, Hron T, et al., Antiviral activity and adaptive evolution of avian tetherins. Journal of virology. 2020; 94(12):e00416-20). Briefly, the aliquot of the supernatant was mixed with Triton X-100-containing lysis buffer, phage MS2 RNA as a template for reverse transcriptase was added, and reverse-transcriptase activity was analysed using quantitative RT-PCR with MS2-specific primers and probe. RT activity as arbitrary units (a.u.) is plotted (Y-axis). Mean of three technical replicates is presented. **c** Infectious particles produced to the supernatant by three variants of Syncytin-1. Two days post-transfection, the supernatant of the pMCAS(Sync1-MS)dsRed- (red), pMCAS(3Flag-Sync1-MS)dsRed- (green) or pMCAS(Sync1-MSC16)dsRed- (blue) transfected cells was collected, filtered through 0.45 µm and transferred to cells expressing FuTraP-hASCT2-wt. Three days after infection, the frequency of infected (dsRed-positive) cells was analysed by flow cytometry (Y-axis). Mean of two parallel infections is presented.

**Additional file 6: Fig. S6. **Cell–cell fusion induced by the interaction of Syncytin-1 with ASCT2 as visualised by microscopy. Non-modified DF-1 or FuTraP-hASCT2-wt cells were seeded and either transfected with pMCAS(3Flag-Sync1-MS)dsRed (the second and fourth panels) or mock-transfected (the first and third panels). Individual channels are shown in grayscale and depicts the cells expressing FuTraP proteins according to iRFP713 fluorescence (coloured in red in merged image) and AcGFP (coloured in cyan in merged image); cells expressing Syncytin-1 can be identified by dsRed fluorescence (coloured in yellow in merged image). The nuclei were stained with DAPI (coloured in blue in merged image). Colourised composites of all channels are enlarged to illustrate the separated single-nuclei cells or the induced multi-nucleated syncytia (lower panel). Cells fixed by 4% paraformaldehyde and mounted in Mowiol + Dapi solution were visualised by a Leica DM6000 microscope (with 63 × /1.4 NA objective). The maximum intensities in 12-planes Z-stack were projected into a single composite image and the contrast was enhanced in ImageJ. The yellow scale bars represent 10 μm.

**Additional file 7: **Movie. Syncytin-1-induced fusion captured by time-lapse microscopy. DF-1/FuTraP-hASCT2 cells were seeded in a glass-bottom 35-mm dish and transfected with pMCAS(3Flag-Sync1-MS)dsRed. Five hours after transfection, the media was exchanged to FluoroBrite DMEM (Thermofisher) supplemented with 4% foetal bovine serum, 4% bovine serum, and 1% chicken serum. Images of cells were taken every 20 min by Andor Dragonfly 503 confocal spinning disc microscope (with a 40 × /1.1 NA objective and a climate chamber). Each image was deconvolved using Huygens software. The maximum intensities of each channel in 13-planes Z-stack were projected in ImageJ. The images were further enhanced in Adobe Photoshop Lightroom with non-linear adjustments. The resulting movie was generated in ImageJ and shows, in greyscale, AcGFP fused with ASCT2 (cyan channel, top left), IRES-driven iRFP713 (red channel, bottom left), cells expressing Syncytin-1 and dsRed (yellow channel, top right), and colourised merge of three previous channels (bottom right). The scale bar represents 10 μm.

**Additional file 8: Fig. S7.** Cell surface expression of Syncytin-1. Live DF-1/HiBiT cells transfected with pMCAS(3Flag-Sync1-MS)dsRed (two right dot plots) or mock-transfected (two left dot plots). Cells were labelled with Anti-Flag® M2-FITC antibody (the second and fourth dot plots) and analysed by flow cytometry for dsRed (X-axis) and FITC (Y-axis) fluorescence. Unlabelled controls were analysed in parallel (the first and third dot plots). Compensated dot plots of 10^4^ cells are shown.

**Additional file 9: **Nucleotide sequence of pFuTraP-hEAAT1 in GenBank format. The s﻿equence is compatible with common text editors or can be directly imported to appropriate software (Vector NTi, SnapGene, etc.).

**Additional file 10: **Nucleotide sequence of pFuTraP-ggTvb^S1^ in GenBank format. The sequence is compatible with common text editors or can be directly imported to appropriate software (Vector NTi, SnapGene, etc.).

**Additional file 11: **Nucleotide sequence of pFuTraP-hASCT2-wt in GenBank format. The sequence is compatible with common text editors or can be directly imported to appropriate software (Vector NTi, SnapGene, etc.).

**Additional file 12: **Nucleotide sequence of pFuTraP-hASCT2-d5 in GenBank format. The sequence is compatible with common text editors or can be directly imported to appropriate software (Vector NTi, SnapGene, etc.).

**Additional file 13: **Nucleotide sequence of pFuTraP-hASCT2-d7 in GenBank format. The sequence is compatible with common text editors or can be directly imported to appropriate software (Vector NTi, SnapGene, etc.).

**Additional file 14: **Nucleotide sequence of pFuTraP-hASCT2-d11 in GenBank format. The sequence is compatible with common text editors or can be directly imported to appropriate software (Vector NTi, SnapGene, etc.).

**Additional file 15: **Nucleotide sequence of pFuTraP-hASCT2-d13 in GenBank format. The sequence is compatible with common text editors or can be directly imported to appropriate software (Vector NTi, SnapGene, etc.).

**Additional file 16: **Nucleotide sequence of pFuTraP-hASCT2-d15 in GenBank format. The sequence is compatible with common text editors or can be directly imported to appropriate software (Vector NTi, SnapGene, etc.).

**Additional file 17: **Nucleotide sequence of pFuTraP-hASCT2-d17 in GenBank format. The sequence is compatible with common text editors or can be directly imported to appropriate software (Vector NTi, SnapGene, etc.).

**Additional file 18: **Nucleotide sequence of pFuTraP-hASCT2-d19 in GenBank format. The sequence is compatible with common text editors or can be directly imported to appropriate software (Vector NTi, SnapGene, etc.).

**Additional file 19: **Nucleotide sequence of pFuTraP-hASCT2-d22 in GenBank format. The sequence is compatible with common text editors or can be directly imported to appropriate software (Vector NTi, SnapGene, etc.).

**Additional file 20: **Nucleotide sequence of pFuTraP-hASCT2-N212Q in GenBank format. The sequence is compatible with common text editors or can be directly imported to appropriate software (Vector NTi, SnapGene, etc.).

**Additional file 21: **Nucleotide sequence of pMCAS(Sync1-MSC16)dsRed in GenBank format. The sequence is compatible with common text editors or can be directly imported to appropriate software (Vector NTi, SnapGene, etc.).

**Additional file 22: **Nucleotide sequence of pMCAS(Sync1-MS)dsRed in GenBank format. The sequence is compatible with common text editors or can be directly imported to appropriate software (Vector NTi, SnapGene, etc.).

**Additional file 23: **Nucleotide sequence of pMCAS(3Flag-Sync1-MS)dsRed in GenBank format. The sequence is compatible with common text editors or can be directly imported to appropriate software (Vector NTi, SnapGene, etc.).

**Additional file 24: **Nucleotide sequence of pMCAS(E)dsRed in GenBank format. The sequence is compatible with common text editors or can be directly imported to appropriate software (Vector NTi, SnapGene, etc.).

**Additional file 25: **Nucleotide sequence of pSU(S1)-RBD-IgG in GenBank format. The sequence is compatible with common text editors or can be directly imported to appropriate software (Vector NTi, SnapGene, etc.).

## Data Availability

The datasets supporting the conclusions of this article are included within the article and its additional files.

## Abbreviations

AcGFP: *Aequorea coerulescens* Green Fluorescent Protein
ALV: Avian Leukosis Virus
ASCT: Alanine, Serine, Cysteine Transporter
ECL: Extracellular Loop
EAAT: Excitatory Amino Acid Transporter
FITC: Fluorescein Isothiocyanate
FuTraP: Fusion Transmembrane Protein
HiBiT: High-Affinity NanoBiT
IRES: Internal Ribosome Entry Site
iRFP713: Infrared Fluorescent Protein 713
LgBiT: Large NanoBiT
MAV: Myeloblastosis Associated Virus
MFI: Mean Fluorescence Intensity
NanoBiT: NanoLuc Binary Technology
RBD: Receptor-Binding Domain
RCASBP: Replication-Competent ALV LTR with Splice Acceptor and Bryan Polymerase
RDR: RD114-and-D-type-retrovirus
SLC: Solute Carrier
SU: Surface subunit
sS1: Soluble form of Syncytin-1
TM: Transmembrane subunit
