# Supplementary figures and images for: Heterologous avian system for quantitative analysis of Syncytin-1 interaction with ASCT2 receptor
Source: Retrovirology. 2021 Jun 22;18:15. doi: 10.1186/s12977-021-00558-0 (PMC8220723; doi:10.1186/s12977-021-00558-0)

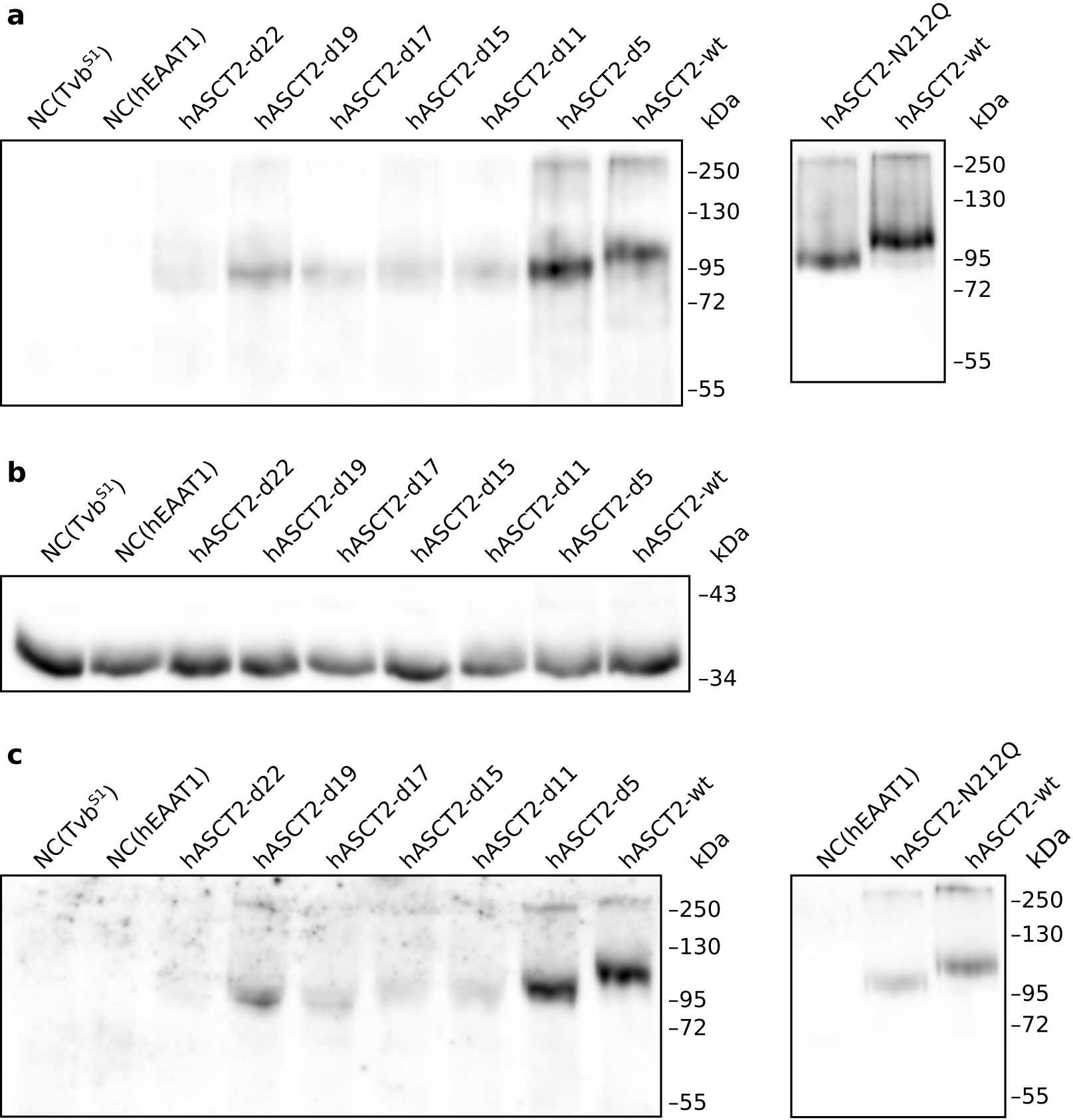

Supplement: Supplementary file 1 — Additional file 1: Fig. S1. Western blot analyses of FuTraP-ASCT2 variants. a Western blot analysis of the FuTraP cell lysates using anti-ASCT2 antibody. Cells were lysed in a solution containing 0.1 M Tris pH 7.6, 7 M urea, 1.5% Triton X-100, mixed with an SDS-containing sample buffer, subjected to 12% SDS-PAGE, and transferred onto PVDF membrane with a semidry system. ASCT2 was detected using a rabbit polyclonal anti-ASCT2 antibody (HPA035240, Sigma-Aldrich) diluted 1:700. Horseradish peroxidase-conjugated secondary goat anti-rabbit antibody (Cell Signaling) and SuperSignal West Femto (ThermoScientific) were used for chemiluminescence detection. The wild-type ASCT2-AcGFP protein has expected molecular weight 84 kDa. b Western blot loading control of the FuTraP cell lysates using anti-GAPDH antibody. The membrane from a was incubated with mouse monoclonal anti-GAPDH antibody (GA1R, ThermoFisher, dilution 1:3000) and detected as in a. c Western blot analysis of the FuTraP cell surface proteins using anti-ASCT2 antibody. Cell surface proteins were isolated using a Pierce Cell Surface Isolation Kit (Thermo Scientific) according to manufacturer’s instructions. Briefly, cell surface proteins on the living cells were biotinylated, isolated with NeutrAvidin Agarose™ and eluted in the SDS lysis buffer. Further analysis was performed as in a. DF-1/LgBiT cells expressing the S1 allele of chicken Tvb (ggTvbS1) or human EAAT1 (hEAAT1) represent the negative controls (NC). kDa molecular weights are indicated. [file 12977_2021_558_MOESM1_ESM.png]

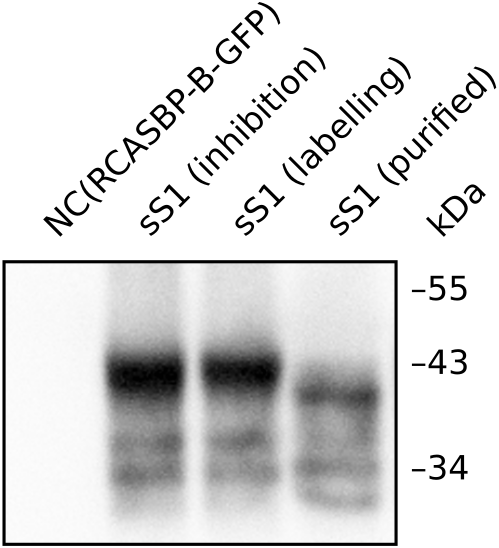

Supplement: Supplementary file 2 — Additional file 2: Fig. S2. sS1-immunoadhesin produced to the supernatant. sS1-immunoadhesin containing the Fc region was immunoprecipitated from the collected supernatant by protein G-Agarose (Pierce, ThermoScientific), mixed with an SDS-containing sample buffer, subjected to 12% SDS-PAGE and transferred onto PVDF membrane with a semidry system. sS1-immunoadhesin was detected using horseradish peroxidase-conjugated goat anti-rabbit IgG antibody (Cell Signaling) and SuperSignal West Femto (ThermoScientific). Separately collected supernatants containing sS1 (as well as RCASBP(B)) that were used for labelling of FuTraP-expressing cells, for inhibition of infection or cell–cell fusion or for purification, were analysed. In contrast to sS1 in the supernatant, the purified sS1 (mAbTrap Kit, Cytiva) was not functional in cell labelling or inhibition experiments and was not used further. Mock control supernatant containing the RCASBP(B)GFP only was used as a negative control (NC). [file 12977_2021_558_MOESM2_ESM.png]

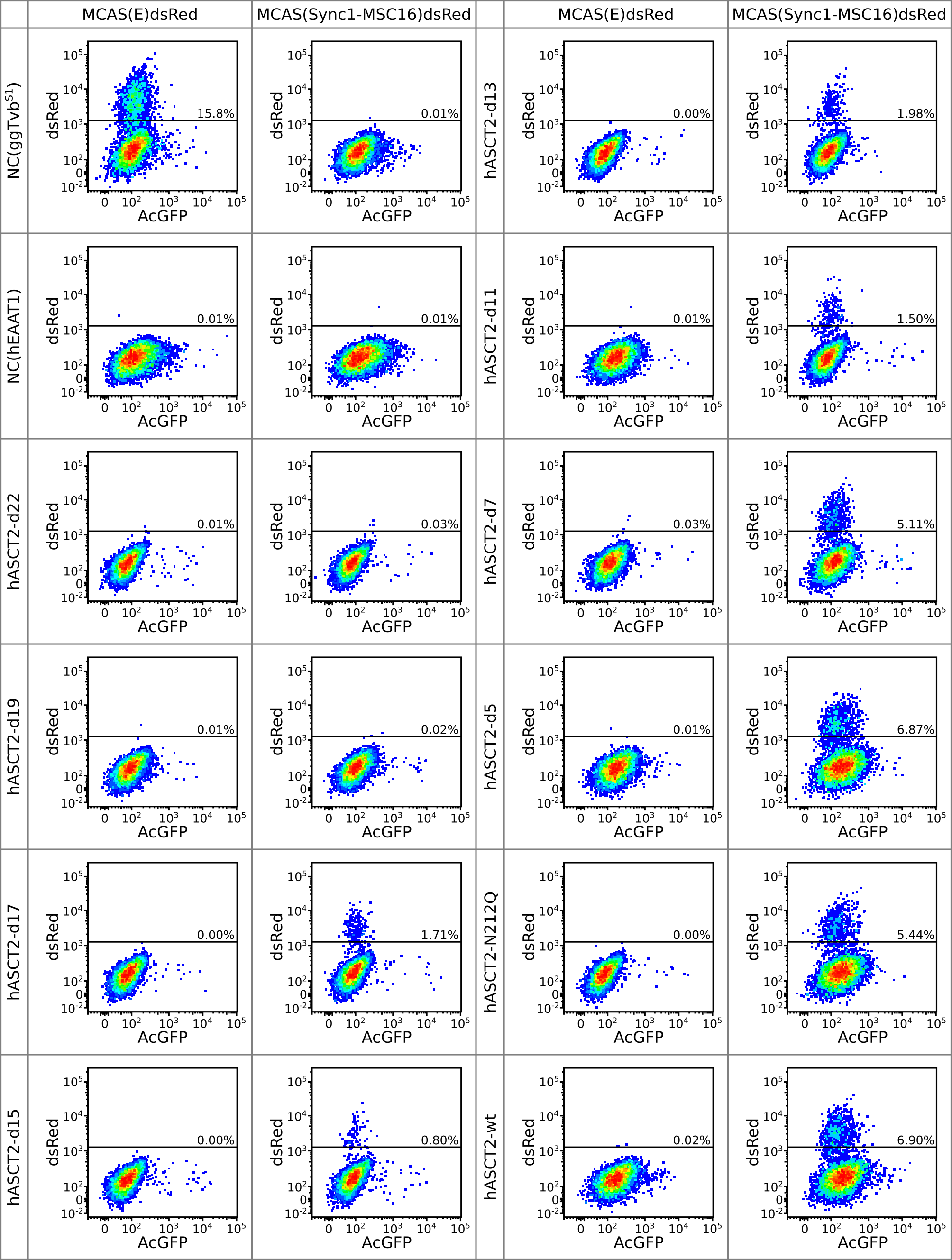

Supplement: Supplementary file 3 — Additional file 3: Fig. S3. Infection of cells expressing FuTraP-ASCT2 variants with Syncytin-1-enveloped virus. Cells modified with variants of FuTraP were infected with MCAS(Sync1-MSC16)dsRed or mock-infected with MCAS(E)dsRed and analysed by flow cytometry. Representative dot plots of 104 cells are shown. The X-axis depicts the AcGFP fluorescence, Y-axis depicts the dsRed fluorescence. The fraction of infected (dsRed-positive) cells was gated. Negative control (NC) is represented by FuTraP-hEAAT1 or FuTraP-ggTvaS1. [file 12977_2021_558_MOESM3_ESM.png]

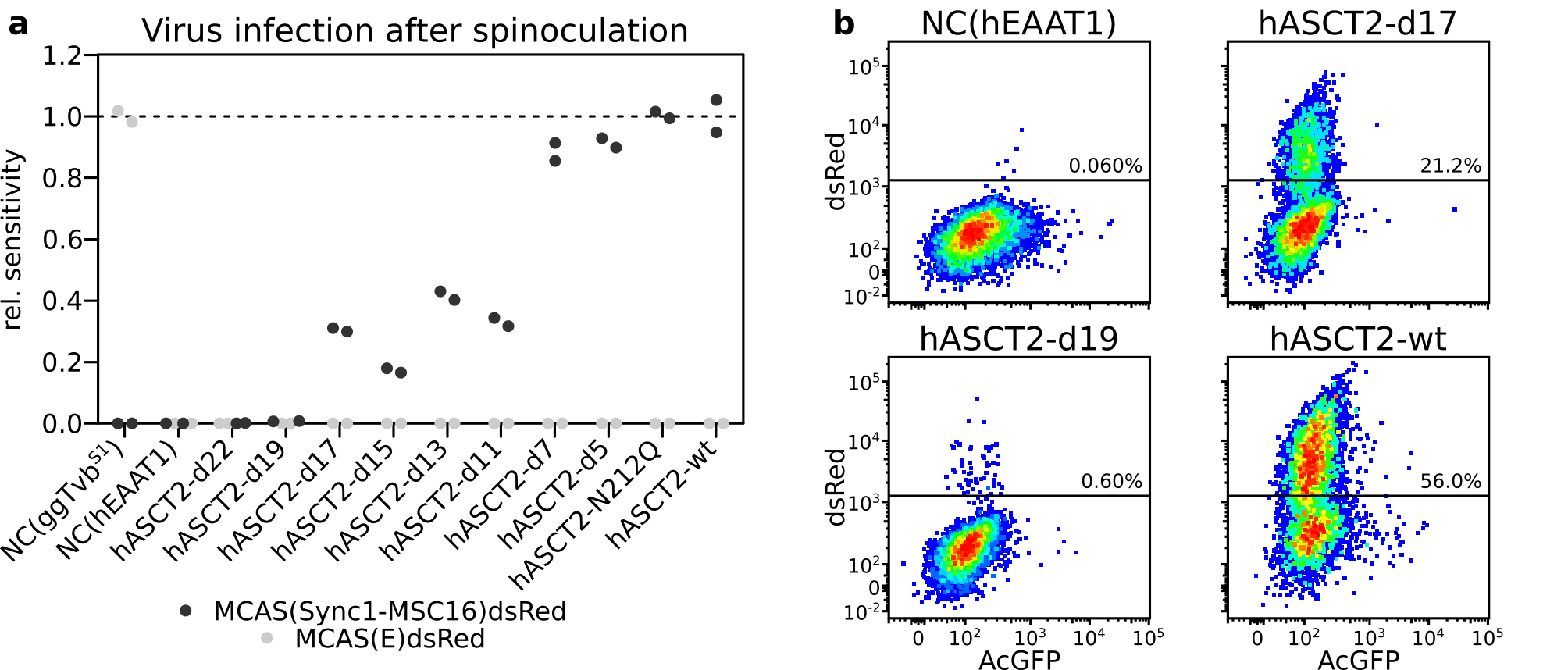

Supplement: Supplementary file 4 — Additional file 4: Fig. S4. Spinoculation of cells expressing FuTraP-ASCT2 variants with Syncytin-1-enveloped virus. a Spinoculation of cells expressing variants of FuTraP with viruses enveloped by Syncytin-1 (black) or Env(E) (gray). Spinoculation was performed as follows: 25 × 103 cells in a 48-well plates were centrifuged with 250 μl of virus supernatant in the presence of 8 μg/ml of polybrene, for 2 h at 1200 × g at 25 °C and the supernatant was replaced with fresh media after the centrifugation. Three days post-infection, the cells were fixed in 1–2% paraformaldehyde (final concentration), analysed by flow cytometry, and the fraction of infected cells was detected by dsRed fluorescence. Sensitivity to viral infection was normalised to the wild-type FuTraP-hASCT2-wt (Y-axis). Results of the representative experiment performed in biological duplicates are plotted. b Representative dot plots of Syncytin-1-enveloped virus spinoculation. FuTraP-hASCT2-wt, FuTraP-hASCT2-d17, FuTraP-hASCT2-d19 and FuTraP-hEAAT1 (NC) cells after spinoculation and flow cytometry analysis are depicted for illustration. The X-axis depicts the AcGFP fluorescence, Y-axis depicts the dsRed fluorescence. The fraction of infected (dsRed-positive) cells was gated. 104 cells are shown. [file 12977_2021_558_MOESM4_ESM.png]

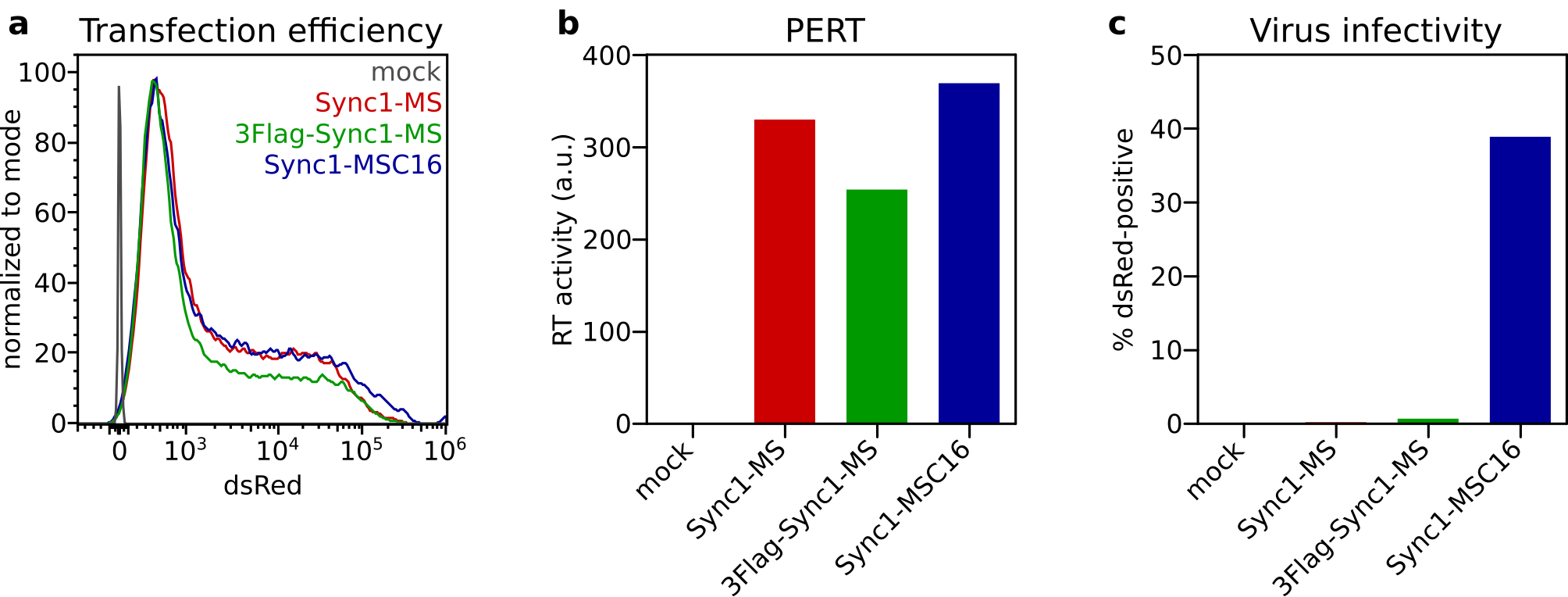

Supplement: Supplementary file 5 — Additional file 5: Fig. S5. Infectivity of Syncytin-1-enveloped viruses. a Transfection efficiency of three variants of Syncytin-1 plasmids. Seeded DF-1 cells were transfected by pMCAS(Sync1-MS)dsRed (red), pMCAS(3Flag-Sync1-MS)dsRed (green) or pMCAS(Sync1-MSC16)dsRed (blue). Two days post-transfection, the supernatant was collected and the cells were analysed by flow cytometry. X-axis depicts the dsRed fluorescence. b Reverse transcriptase activity produced to the supernatant by three variants of Syncytin-1. Two days post-transfection, the supernatant of the pMCAS(Sync1-MS)dsRed- (red), pMCAS(3Flag-Sync1-MS)dsRed- (green) or pMCAS(Sync1-MSC16)dsRed- (blue) transfected cells was analysed for production of reverse transcriptase activity to the supernatant by means of product-enhanced reverse transcriptase (PERT) assay (as published in Krchlikova V, Fabryova H, Hron T, et al., Antiviral activity and adaptive evolution of avian tetherins. Journal of virology. 2020; 94(12):e00416-20). Briefly, the aliquot of the supernatant was mixed with Triton X-100-containing lysis buffer, phage MS2 RNA as a template for reverse transcriptase was added, and reverse-transcriptase activity was analysed using quantitative RT-PCR with MS2-specific primers and probe. RT activity as arbitrary units (a.u.) is plotted (Y-axis). Mean of three technical replicates is presented. c Infectious particles produced to the supernatant by three variants of Syncytin-1. Two days post-transfection, the supernatant of the pMCAS(Sync1-MS)dsRed- (red), pMCAS(3Flag-Sync1-MS)dsRed- (green) or pMCAS(Sync1-MSC16)dsRed- (blue) transfected cells was collected, filtered through 0.45 µm and transferred to cells expressing FuTraP-hASCT2-wt. Three days after infection, the frequency of infected (dsRed-positive) cells was analysed by flow cytometry (Y-axis). Mean of two parallel infections is presented. [file 12977_2021_558_MOESM5_ESM.png]

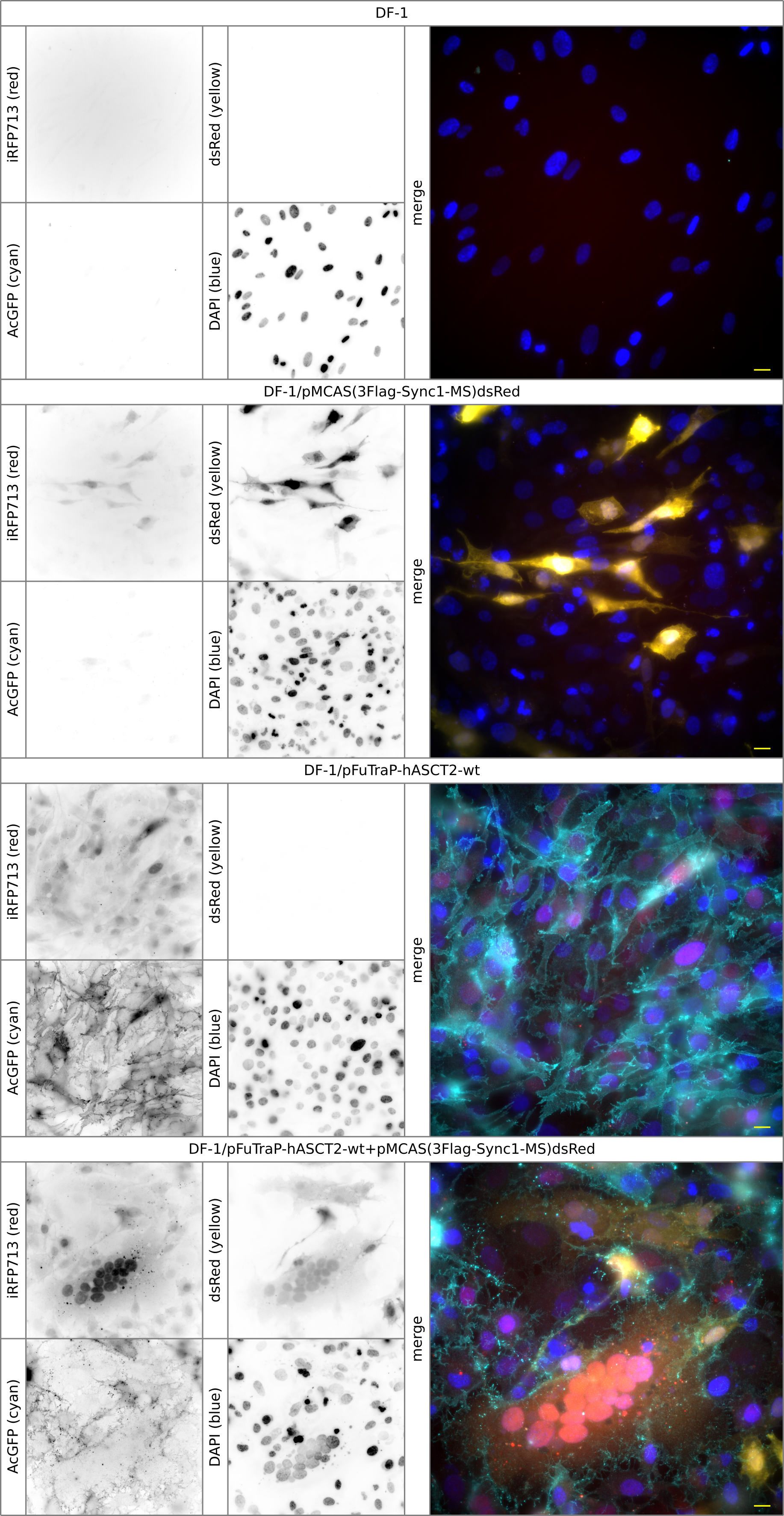

Supplement: Supplementary file 6 — Additional file 6: Fig. S6. Cell–cell fusion induced by the interaction of Syncytin-1 with ASCT2 as visualised by microscopy. Non-modified DF-1 or FuTraP-hASCT2-wt cells were seeded and either transfected with pMCAS(3Flag-Sync1-MS)dsRed (the second and fourth panels) or mock-transfected (the first and third panels). Individual channels are shown in grayscale and depicts the cells expressing FuTraP proteins according to iRFP713 fluorescence (coloured in red in merged image) and AcGFP (coloured in cyan in merged image); cells expressing Syncytin-1 can be identified by dsRed fluorescence (coloured in yellow in merged image). The nuclei were stained with DAPI (coloured in blue in merged image). Colourised composites of all channels are enlarged to illustrate the separated single-nuclei cells or the induced multi-nucleated syncytia (lower panel). Cells fixed by 4% paraformaldehyde and mounted in Mowiol + Dapi solution were visualised by a Leica DM6000 microscope (with 63 × /1.4 NA objective). The maximum intensities in 12-planes Z-stack were projected into a single composite image and the contrast was enhanced in ImageJ. The yellow scale bars represent 10 μm. [file 12977_2021_558_MOESM6_ESM.png]

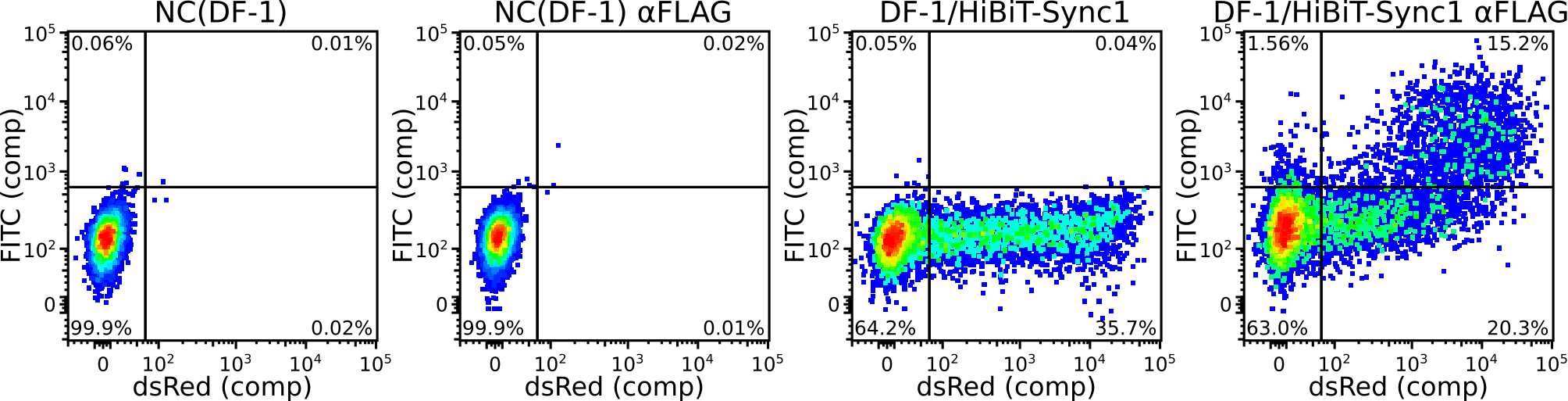

Supplement: Supplementary file 8 — Additional file 8: Fig. S7. Cell surface expression of Syncytin-1. Live DF-1/HiBiT cells transfected with pMCAS(3Flag-Sync1-MS)dsRed (two right dot plots) or mock-transfected (two left dot plots). Cells were labelled with Anti-Flag® M2-FITC antibody (the second and fourth dot plots) and analysed by flow cytometry for dsRed (X-axis) and FITC (Y-axis) fluorescence. Unlabelled controls were analysed in parallel (the first and third dot plots). Compensated dot plots of 104 cells are shown. [file 12977_2021_558_MOESM8_ESM.png]
